# Molecular Genetic Architecture of Monogenic Pediatric IBD Differs from Complex Pediatric and Adult IBD

**DOI:** 10.3390/jpm10040243

**Published:** 2020-11-26

**Authors:** Gregor Jezernik, Dušanka Mičetić-Turk, Uroš Potočnik

**Affiliations:** 1 Faculty of Medicine, University of Maribor, Taborska Ulica 8, 2000 Maribor, Slovenia; gregor.jezernik1@um.si (G.J.); dusanka.micetic@um.si (D.M.-T.); 2 Faculty of Chemistry and Chemical Engineering, University of Maribor, Smetanova Ulica 17, 2000 Maribor, Slovenia

**Keywords:** pediatric Crohn’s disease, indeterminate colitis, Gene Ontology, immunologic deficiency syndromes

## Abstract

Inflammatory bowel disease (IBD) manifests as a complex disease resulting from gene–environment interactions or as a monogenic disease resulting from deleterious mutations. While monogenic IBD is predominantly pediatric, only one-quarter of complex IBD is pediatric. In this study, we were the first to systematically compare genetic architecture between monogenic and complex pediatric and adult IBD on genetic and molecular pathway levels. Genes reported as causal for monogenic pediatric IBD and related syndromes and as risk factors for pediatric and adult complex IBD were analyzed using CytoScape and ClueGO software tools to elucidate significantly enriched Gene Ontology (GO) terms. Despite the small overlap (seven genes) between monogenic IBD genes (85) and complex IBD loci (240), GO analysis revealed several enriched GO terms shared between subgroups (13.9%). Terms Th17 cell differentiation and Jak/STAT signaling were enriched in both monogenic and complex IBD subgroups. However, primary immunodeficiency and B-cell receptor signaling pathway were specifically enriched only for pediatric subgroups, confirming existing clinical observations and experimental evidence of primary immunodeficiency in pediatric IBD patients. In addition, comparative analysis identified patients below 6 years of age to significantly differ from complex pediatric and adult IBD and could be considered a separate entity.

## 1. Introduction

Inflammatory bowel disease (IBD) is an immune-mediated disease of the gastrointestinal tract. Current understanding of complex IBD pathogenesis highlights incorrect and insufficient immune responses to microbes in the gut mucosa that arise in a genetically susceptible host. First IBD symptoms most commonly occur in adulthood; however, about one-quarter of IBD patients are affected before the age of 20 [1,2]. In Slovenia, the prevalence of pediatric Crohn’s disease (CD) patients is 8.6 per 100,000 per year [3]. Pediatric IBD is usually resistant to treatment and causes extensive inflammation of the gastrointestinal tract [4,5]. Additional complications of pediatric-onset IBD are developmental delay and malnourishment due to poor nutrient absorption [6], as well as immune deficiencies [7]. Recurrent gastrointestinal infections also require remediation of the gut flora [8]. These differences and others influenced the development of pediatric diagnostic and treatment algorithms (European Society for Paediatric Gastroenterology Hepatology and Nutrition—ESPGHAN guidelines) [9,10,11,12,13]. Pediatric IBD is more commonly classified as CD than ulcerative colitis (UC); however, a significant fraction is classified as indeterminate colitis [5,14]. In addition, approximately one-third of all immune-mediated pediatric monogenic disorders present with IBD-like symptoms, making accurate diagnosis without genetic testing difficult [15].

Pediatric-onset IBD is thought to have a greater genetic component compared to adult-onset IBD due to lower cumulative exposure to environmental factors. Rare monogenic IBD and IBD-like syndromes, thus, present the genetic extreme where a single highly pathogenic variant causes IBD symptoms. As such, pediatric-onset IBD represents a spectrum ranging from extreme monogenic variants to adolescent complex variants. In an Italian cohort, a monogenic syndrome was confirmed in 75% of patients younger than 1 month, but only in 12–19% of patients younger than 6 years [16], demonstrating the early age of onset in extreme genetic cases. Recent studies estimate that approximately 3% [17] to 6% [18] of all pediatric IBD cases are monogenic disorders.

The genetic component of adult IBD has been extensively studied. To date, genome-wide association studies (GWASs) and post-GWAS deep resequencing studies have identified 240 IBD-associated loci [19,20,21,22,23,24,25]. Gene Ontology (GO) analysis of these IBD associated loci has given insight into biological processes which underlie IBD pathogenesis, highlighting the importance of host–microbe interactions.

Meanwhile, few GWASs of pediatric-onset IBD have been performed to date. Early pediatric IBD GWAS revealed two loci associated specifically with early-onset CD (*TNFRSF6* and *PSMG1*); however, later studies revealed that the loci are also associated with adult-onset CD [26]. Later, whole-exome sequencing followed by replication GWAS revealed an overlap of genetic loci associated with pediatric and adult onset IBD in Poland, as well as a significant accumulation of rare and deleterious variants in affected children [27]. A large-scale GWAS of IBD also identified three loci linked to a lower age of onset [28]. GWASs had success in identifying loci with enhanced contribution in pediatric-onset UC [29]. This suggests that the pathogenesis of pediatric and adult inflammatory bowel disease does not differ much. However, GWASs are by design unlikely to detect variants suspected to be causative of pediatric IBD, i.e., low-frequency intermediate effect variants [30]. Furthermore, similar comparisons between monogenic and complex variants of other diseases revealed a major difference in causative genes [31].

To determine what larger sets of genes have in common and how they interact with each other, Gene Ontology (GO) analysis is employed. According to existing knowledge of gene function, GO databases have been constructed and are constantly updated and expanded. Using these highly integrated databases, it is possible to determine what processes underlie pathogenesis. To date, no study has performed GO analysis of genes causative for pediatric IBD and IBD-like disorders or compared them to complex pediatric and adult IBD. GO analysis could reveal similarities and differences between pediatric-onset and adult-onset IBD and help elucidate processes which contribute the most to early onset IBD. Understanding the molecular genetic architecture underlying pediatric IBD pathogenesis can open opportunities for novel therapeutic approaches and drug development, as well as optimization of existing treatment strategies [32]. Furthermore, knowledge of the genetic landscape that shapes pediatric-onset IBD would allow for gene panel optimization to reduce cost of genetic testing and increase its availability. To this end, we gathered genes with variants causative for pediatric IBD and IBD-like disorders to elucidate enriched biological processes using GO analysis.

## 2. Materials and Methods

To obtain scientific literature relevant to the genetics of pediatric IBD and IBD-like syndromes, we performed a search of the PubMed databases using following search terms:

(“Inflammatory Bowel Diseases” [Mesh] OR “Pediatric Crohn’s disease” [Supplementary Concept]) AND (“Pediatrics” [Mesh] OR “very early onset” OR “Mendelian”) AND (“Genetics” [Mesh] OR “gene”).

Then, we expanded our literature search to Online Mendelian Inheritance in Man (OMIM), National Center for Biotechnology Information Genetic Testing Registry (NCBI GTR), and ORPHANET databases using following terms: pediatric inflammatory bowel disease, pediatric indeterminate colitis, pediatric Crohn’s disease, and pediatric ulcerative colitis.

Studies describing pediatric IBD and IBD-like syndromes and associated genes were included on the basis of the following criteria: disorder is described or presented itself as inflammatory bowel disease, Crohn’s disease, ulcerative colitis or indeterminate colitis; disorder first affects patients younger than 18 years; disorder has an accurately described disease onset; disorder is monogenic or shown to be highly penetrant (>90%); disorder is causally linked to a known genetic locus.

IBD and IBD-like syndromes were then divided into subsets according to age of onset according to Montreal classification and the Pediatric Paris modification [7]. IBD and IBD-like syndromes with a reported age of onset spanning two or more subgroups were attributed to all relevant subgroups. As such, pediatric IBD subgroups of IBD and IBD-like syndromes and their causal genes were defined as follows: neonatal IBD with age of onset <28 days (NEO-IBD); infantile IBD with age of onset <2 years (INF-IBD); very-early-onset IBD with age of onset <6 years (VEO-IBD); early-onset IBD with age of onset <10 years (EO-IBD); pediatric IBD with age of onset <18 years (PED-IBD).

**Figure 1:**
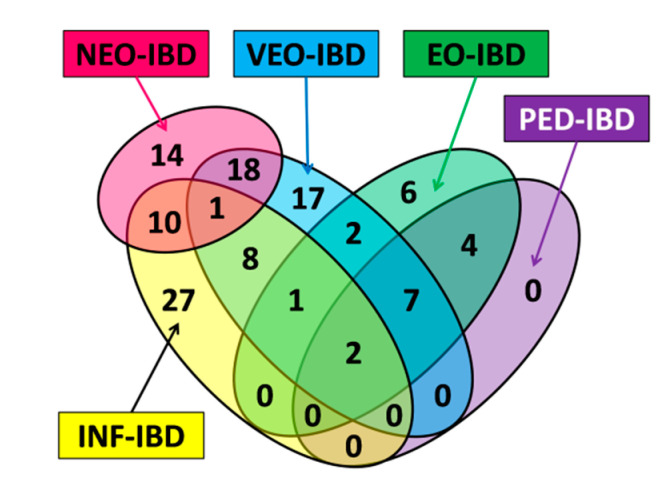
Venn diagram of monogenic IBD (inflammatory bowel disease) and IBD-like syndrome gene subgroup overlaps. NEO-IBD: neonatal IBD with age of onset < 28 days; INF-IBD: infantile IBD with age of onset < 2 years; VEO-IBD: very-early-onset IBD with age of onset < 6 years; EO-IBD: early-onset IBD with age of onset < 10 years; PED-IBD: pediatric IBD with age of onset < 18 years.

Additional pediatric IBD subgroups were defined by combining primary subgroups. The combined subgroups UN6 (shortened for “under 6 years of age”) and OV6 (shortened for “over 6 years of age” are based on the threshold age of onset at 6 years. Thus, “UN6” contains all variants associated with an age of onset below 6 years of age while “OV6” contains all variants associated with an age of onset above 6 years of age. A detailed definition of subgroups is summarized in Table 1 and illustrated in Figure 1.

To compare the genetic architecture of pediatric and adult IBD, we also constructed two subgroups of adult IBD-associated genes, one based on current genetic data (ADULT-1) and another based on the GO term inflammatory bowel disease (ko05321). Normally, ADULT-1 would be based on two major genome-wide association studies (GWASs) of IBD, which identified 163 IBD risk loci in 2012 [19] and an additional 38 loci in 2015 [23], and recent studies that increased the total identified IBD risk loci to 240 [20,21,22,24]. However, since more than 80% of IBD-associated single-nucleotide polymorphisms (SNPs) identified in GWASs are located in noncoding regions, between genes, or even in “gene desert” regions, pinpointing the true causal gene has proven difficult. Since GO analysis requires a definite causal gene, we compiled a list of IBD-associated genes for GO analysis on the basis of 240 adult-onset IBD-associated loci and their expressive quantitative trait loci (eQTL) data. The reported causal genes and expressive quantitative trait loci (eQTL) traits were reviewed for all 240 loci using data from comprehensive IBD loci fine-mapping studies [33,34], the Ensembl genome browser (for both LD distance-based gene search and eQTL data-based fine mapping) [35], and/or the Genotype-Tissue Expression (GTEx) Portal [36]. Lastly, only genes with either confirmed causal protein-disrupting variants (missense or frameshift variants) or matching eQTL data were included in the complex adult IBD group definition (Table S1, Supplementary Materials.

The second adult IBD gene list, ADULT-2, was based exclusively on the GO term inflammatory bowel disease (ko05321) as defined by Kyoto Encyclopedia of Genes and Genomes (KEGG) according to experimental evidence of genes involved in the pathological processes in the gastrointestinal tract of IBD patients [37]. Terms K06752 (*MHC2*), K10784 (*TRAV*), and K10785 (*TRBV*) were excluded as they do not specify a single gene, but a group of genes or a gene family (Table S2, Supplementary Materials).

Then, Gene Ontology analysis was performed using software package CytoScape 3.6.1. [38] with the integrated application ClueGO v2.5.1 [39]. ClueGO analysis was performed using databases last updated November 2019 and using the following parameters and selected options:

Ontology/pathways selected: biological process, cellular component, immune system process, molecular function, and KEGG;

Evidence selected: *All_Experimental*;
Network specificity: medium (default);
Use GO term fusion: not selected;
Show only pathways with pV < 0.05 (default);
Advanced term/pathway selection options: none selected;
Statistical options: none selected;
Grouping options: none selected.

## 3. Results

### 3.1. Literature Search

We included 36 relevant articles in the final pediatric monogenic Gene Ontology database (Table S3, Supplementary Materials). Together, these 36 articles cover approximately 4200 analyzed patients with monogenic IBD and IBD-like syndromes. For adult complex IBD genes, six studies were included [19,20,21,22,23,24], the most recent and comprehensive of which analyzed 34,213 controls and 33,498 IBD cases using ImmunoChip data from The International IBD Genetics Consortium (IIBDGC) [24]. We also included two studies analyzing pediatric complex IBD which analyzed 136 [26] and 1495 [27] cases, as well as one study of complex IBD reporting loci associated with lower age of onset which analyzed 34,819 IBD patients [28], including pediatric patients.

### 3.2. Gene Subgroup Definition

The final list of IBD and IBD-like monogenic disorders and associated genes is displayed in (Table S4 Supplementary Materials). Complex adult IBD gene subgroup ADULT-1 was defined on the basis of refined complex IBD loci data (Table S1, Supplementary Materials), while ADULT-2 was defined using the KEGG database as described previously (Table S2, Supplementary Materials).

### 3.3. Gene Ontology Analysis Results

**Figure 2:**
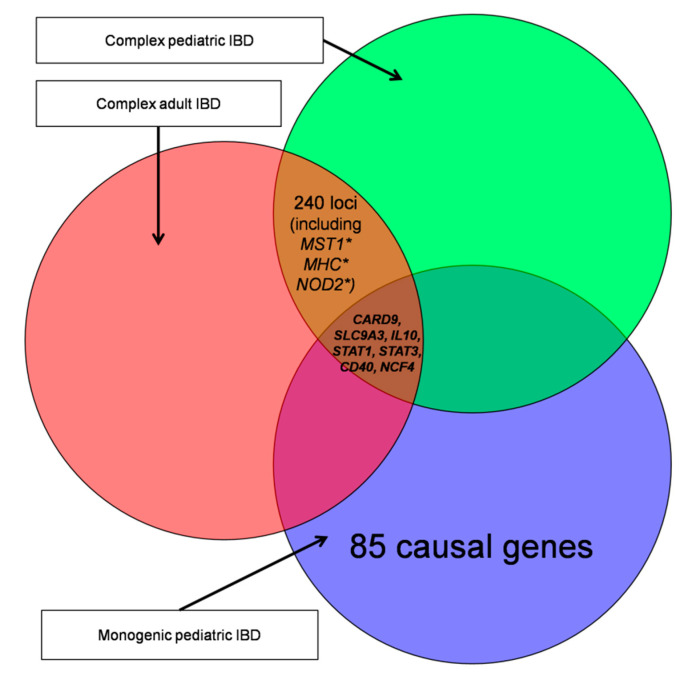
Venn diagram of monogenic pediatric inflammatory bowel disease IBD, complex pediatric IBD, and complex adult IBD gene groups and their overlaps. *MST1*: macrophage stimulating 1 gene, *MHC*: major histocompatibility complex genes; *NOD2*: nucleotide binding oligomerization domain containing 2; *CARD9*: caspase recruitment domain family member 9 gene; *SLC9A3*: solute carrier family 9 member A3 gene; *IL10*: interleukin 10 gene; *STAT1*: signal transducer and activator of transcription 1 gene; *STAT3*: signal transducer and activator of transcription 3 gene; *CD40*: CD40 molecule gene; *NCF4*: neutrophil cytosolic factor 4 gene.

Comparisons of pediatric-onset IBD (ALL pediatric subsets; 85 genes) and adult-onset IBD genes (ADULT-1 subset; 240 loci) revealed a small overlap of genes *SLC9A3, IL10, STAT1, CARD9, STAT3, CD40*, and *NCF4*. Overlap of loci associated with complex adult IBD, complex pediatric IBD, and monogenic pediatric IBD is displayed in Figure 2.

**Figure 3:**
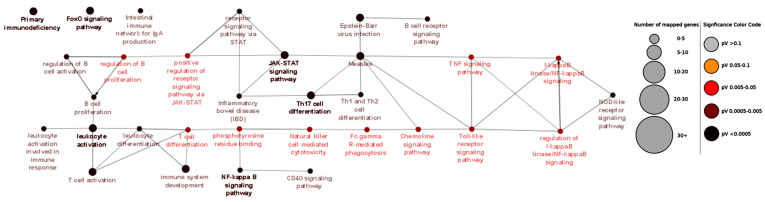
Enrichment map for Gene Ontology of all monogenic pediatric inflammatory bowel disease (IBD) genes. The figure illustrates the high interconnectivity of enriched gene ontology (GO) terms in the complete monogenic IBD gene subset (ALL), but also that genes associated with primary immunodeficiency are not linked with any other GO term. Terms with bold font labels are top GO terms within their analyzed subgroup, as presented by ClueGO default settings. All GO terms shown are statistically significant (*p* < 0.05) after Bonferonni correction. Th17: T helper 17; CD40: CD40 molecule; STAT: signal transducer and activator of transcription proteins; TNF: Tumor necrosis factor; NOD: Nucleotide-binding oligomerization domain-containing protein.

**Figure 4:**
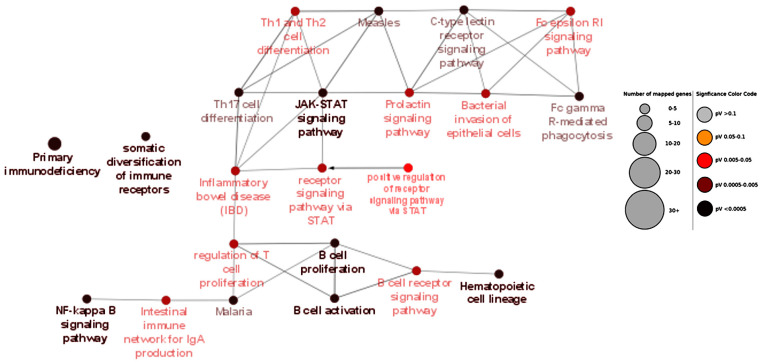
Enrichment map for Gene Ontology of infantile-onset pediatric IBD genes. The figure presents a closer look at the enriched gene ontology (GO) terms in the infantile onset IBD gene subset INF. STAT: signal transducer and activator of transcription proteins; Fc epsilon RI: FcεRI protein; lgA: Immunoglobulin A.

First, the Gene Ontology of subgroups was analyzed using ClueGO. The primary subgroup analysis of NEO-IBD, (<28 days), INF-IBD (<2 years), VEO-IBD (<6 years), EO-IBD (<10 years), and PED-IBD < 18 years) revealed several enriched GO terms. Most importantly, the GO term *primary immunodeficiency* was enriched in infantile IBD subset INF (*p* = 1.22 × 10^−22^), very-early-onset IBD subset VEO (*p* = 2.30 × 10^−9^), and early-onset IBD subset EO (*p* = 3.13 × 10^−5^). An enrichment plot for Gene Ontology analysis of all monogenic pediatric IBD genes (subset ALL) is shown in Figure 3. Additionally, Figure 4 depicts the enrichment plot for Gene Ontology results of the INF subset. Significant results of subset GO analysis are also summarized in Table 2, whereas all results are shown in (Table S5 Supplementary Materials).

To further investigate the role of primary immunodeficiency in complex pediatric and adult IBD, we also performed Gene Ontology analysis of the adult gene subset ADULT-1, and then repeated the analysis excluding all monogenic pediatric IBD genes. Gene Ontology analysis showed that *primary immunodeficiency* is not enriched in the complete ADULT-1 or in the ADULT-1 subset without monogenic pediatric IBD genes. Results for both analyses can be found in (Table S5 Supplementary Materials).

**Figure 5:**
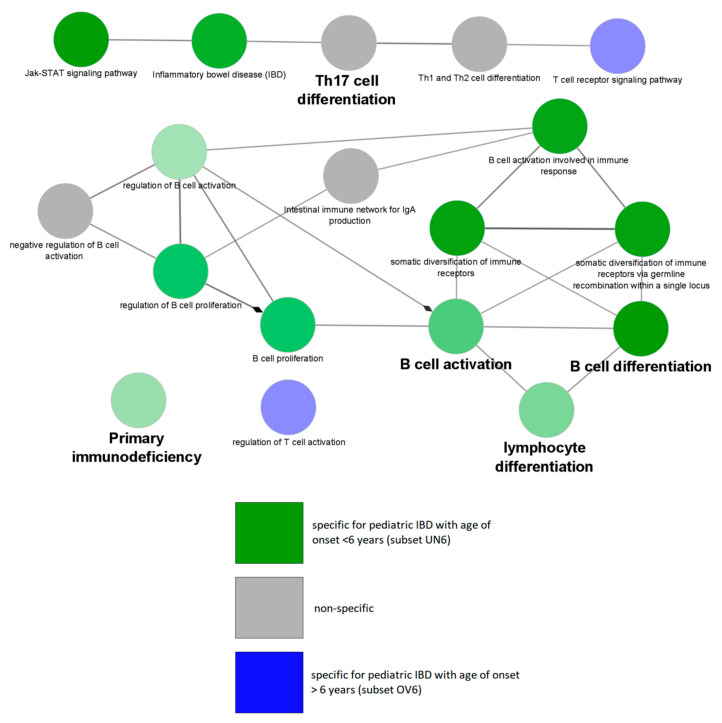
Enrichment map depicting specificity of gene ontology GO terms for comparative Gene Ontology analysis of monogenic pediatric inflammatory bowel disease (IBD) gene subsets based on age of onset, specifically under 6 years of age (UN6) and over 6 years of age (OV6). Green terms are specific for subset UN6, blue terms are specific for subset OV6, and gray terms are nonspecific. Color intensity depicts how specific each term is for a subset. Terms with bold font labels are top GO terms within their analyzed subgroup, as presented by ClueGO default settings. All GO terms shown are statistically significant (*p* < 0.05) after Bonferonni correction.

Next, similarities and differences of subsets were analyzed using comparative Gene Ontology. Interestingly, the comparative analysis of UN6 and OV6 showed that GO terms primary immunodeficiency (*p* = 8.82 × 10^−32^), B-cell receptor signaling pathway (*p* = 1.51 × 10^−7^), and Jak/STAT signaling pathway (*p* = 1.57 × 10^−7^) were enriched and specific for UN6 (age of onset <6 years) while T-cell receptor signaling pathway (*p* = 1.99 × 10^−9^) and regulation of T-cell activation (*p* = 8.60 × 10^−6^) were specific for OV6 (age of onset >6 years). The comparative Gene Ontology analysis of UN6 versus OV6 is visualized in Figure 5.

**Figure 6:**
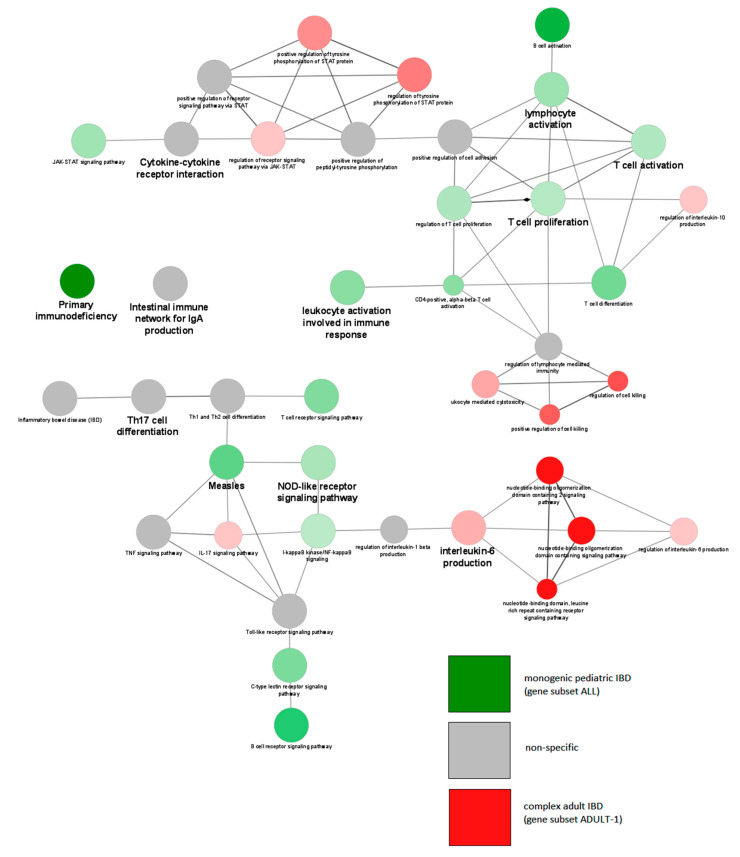
Enrichment map depicting specificity of GO terms for comparative Gene Ontology analysis of monogenic pediatric inflammatory bowel disease (IBD) genes (subset ALL) and complex adult IBD genes (ADULT-1). Green terms are specific for monogenic pediatric genes, red terms are specific for complex adult IBD, and gray terms are nonspecific. Color intensity depicts how specific each term is for a subset. Terms with bold font labels are top GO terms within their analyzed subgroup, as presented by ClueGO default settings. All GO terms shown are statistically significant (*p* < 0.05) after Bonferonni correction.

Importantly, comparison of monogenic pediatric IBD (gene subset ALL) versus complex adult IBD (subset ADULT-1) confirmed the GO term primary immunodeficiency to be highly specific for monogenic pediatric IBD. Results of ALL versus ADULT-1 subset comparative Gene Ontology analysis are graphically represented in Figure 6. Results of all comparative analyses are summarized in Table 3, while full results are shown in (Table S6 Supplementary Materials). Comparative Gene Ontology analysis of gene subset ALL versus the gene subset ADULT-2 based on the inflammatory bowel disease *(IBD)* GO term was not feasible to visualize using an enrichment plot due to the high number of terms specific to the subset ADULT-2; instead, results are summarized in Table 3, while full results are shown in (Table S6 Supplementary Materials).

## 4. Discussion and Conclusions

Our study highlights processes underlying the genetic architecture of pediatric IBD and IBD-like syndromes, as well as differences and similarities of pediatric and adult onset IBD. Moreover, our bioinformatics results highlight primary immunodeficiency as a key feature in the majority of early-onset monogenic IBD, adding additional confirmation to existing clinical observations and experimental evidence of immunodeficiency in children with very-early-onset IBD and IBD-like pediatric syndromes.

Surprisingly, our literature search revealed there is little overlap between the 85 monogenic pediatric IBD genes and the 240 complex adult IBD loci (specifically ADULT-1), as only seven genes were identified in the overlap (*SLC9A3, IL10, STAT1, CARD9, STAT3, CD40,* and *NCF4*), representing 8.1% of the monogenic pediatric IBD genes and 2.9% of complex adult IBD genes. However, despite the small gene overlap between both groups, comparative analysis revealed a much larger overlap (13.9%) of biological processes underlying monogenic pediatric IBD genes (ALL subset) and complex adult IBD genes (ADULT-1 subset). Out of significantly enriched 424 GO terms, 59 terms (13.9%) were in overlap. The remaining GO terms were found to be highly specific for either the monogenic pediatric IBD group or the complex adult IBD.

Most importantly, GO term primary immunodeficiency (KEGG:05340) was enriched in several pediatric subgroups. Interestingly, the fraction of genes associated with this GO term decreased and the *p*-value increased as the age of onset threshold increased. Thus, primary immunodeficiency was associated with 32.43% genes in INF-IBD (*p* = 1.22 × 10^−22^), 16.22% in VEO-IBD (*p* = 2.30 × 10^−9^), and 8.11% in EO-IBD (*p* = 3.13 × 10^−5^). This inverse correlation between the fraction of associated genes and age of onset suggests that primary immunodeficiency is a driving factor of pediatric IBD and IBD-like syndromes, especially syndromes which manifest very early. Comparative analysis of combined subgroups additionally elucidated the significance of primary immunodeficiency. Further analysis of combined subgroups (NEO-, INF-, and VEO- vs. EO- and PED-IBD) revealed that primary immunodeficiency is highly specific for disorders with age of onset <6 years. Lastly, comparative analysis with both adult IBD subgroups (ADULT-1 and ADULT-2) revealed that primary immunodeficiency is both statistically significant and specific for pediatric IBD and IBD-like syndromes. Primary immunodeficiency genes were shown to be enriched in the original 163 IBD loci in 2012 [19]; however, our analysis using newest GO term databases showed that the term is no longer enriched for adult IBD. Most importantly, this finding supports clinical observations of primary immunodeficiency in children with very-early-onset IBD (<6 years of age). Two recent studies demonstrated the usefulness of targeted sequencing for screening monogenic IBD and IBD-like syndromes in pediatric IBD and both studies highlighted primary immunodeficiency disorders representing a fraction of pediatric monogenic IBD [40,41]. In addition, a recent pediatric IBD study implied several immune process deficiencies in pediatric IBD, adding further evidence to the role of immunodeficiency in pediatric IBD [42]. The study highlighted the role of misregulated and deficient leukocyte process with GO terms such as positive regulation of immune effector process (GO:0002699) and regulation of leukocyte-mediated immunity (GO:0002703) [42], which overlaps with our results and the hypernym primary immunodeficiency.

Several terms related to B cells and T cells were enriched in several pediatric subgroups. Comparative GO analysis of pediatric subgroups revealed B-cell GO terms to be specifically enriched in subgroups with age of onset <6 years, while T-cell processes were enriched in subgroups with age of onset >6 years. During comparative analysis with adult subgroups, both B-cell and T-cell processes remained specific for pediatric IBD and IBD-like syndromes. These findings suggest that very-early-onset IBD is driven mainly by defects in B-cell processes, while later-onset pediatric IBD is influenced by changes in T-cell processes. Moreover, Th1 and Th2 cell differentiation was enriched in pediatric subsets, but was later shown to be specific for subgroup ADULT-2 but not ADULT-1, suggesting that this process is important for all IBD variants. Furthermore, studies have shown that Th17 cells have a role in IBD pathogenesis [43]. The function and maturation of Th17 cells are affected by the STAT cascade [44]. A comprehensive gene expression study comparing pediatric IBD patients and healthy controls showed three gene clusters to be differentially expressed in pediatric IBD patients [45]. The study’s comparative Gene Ontology analysis revealed GO terms such as glucocorticoid receptor signaling, IL-4 (interleukin-4) signaling, and dendritic cell maturation to be significantly associated with pediatric IBD [45]. In our study, where we performed GO analysis according to genes associated with pediatric IBD via mutations and SNPs, we could not replicate the exact GO terms, although subsequent hypernym/hyponym analysis showed some overlap with terms Th1 and Th2 cell differentiation.

The GO terms Th17 cell differentiation (KEGG:04659), Jak/STAT signaling pathway (KEGG:04630), and *STAT cascade* (GO:0097696) were enriched in several pediatric subgroups. Several other hypernyms and hyponyms of these three GO terms were also significantly enriched, representing an enrichment in the IL-23/IL-17 immune axis. Comparative GO analysis between pediatric subgroups revealed that Jak/STAT signaling pathway and STAT cascade are specific for pediatric IBD with age of onset <6 years, while Th17 cell differentiation is not specific for any particular subgroup. However, later comparative GO analysis with complex IBD subgroups ADULT-1 and ADULT-2 showed these terms to be specific for adult IBD. This was highlighted in the overlap between pediatric and adult IBD genes in *CD40, IL10, STAT1,* and *STAT3,* which are involved in Th17 cell processes. Our results, thus, suggest that there is involvement of Th17 cell processes in both pediatric and adult IBD. However, Th17 cell activity blockage with secukinumab, a human anti-IL-17A monoclonal antibody, proved to be ineffective in treating Crohn’s disease [46]. Furthermore, Th17 cells and IL-17A have been shown to play an important role in mucosal healing [47,48,49,50]. In fact, case reports suggest that IBD-like symptoms appear in psoriasis patients treated with IL-17A blockage [51]. The failure of secikunumab in IBD and the role of Th17 cells in IBD suggests that Th17 activity is not directly pathogenic. Rather, their inactivity, misregulation, or a specific immune deficiency of Th17 cells could lead to pathogenic changes in both pediatric and adult IBD. In addition, Jak inhibitors have shown efficacy in IBD, specifically severe ulcerative colitis [52,53]. Our results suggest that Jak inhibitor indication could also be extrapolated to pediatric IBD with suspected aberrant Jak/STAT signaling.

Unsurprisingly, GO term inflammatory bowel disease (IBD) was enriched. Within the pediatric groups, it was enriched only in NEO and INF subgroups and the combined UN6 group. These results coincide with our observations that there is little overlap between pediatric and adult IBD genes. In addition, comparisons with adult IBD groups revealed that GO term inflammatory bowel disease (IBD) is highly specific for adult IBD groups (both ADULT-1 and ADULT-2), highlighting the molecular differences between pediatric IBD and IBD-like syndromes and adult complex IBD.

The main limitation of our study was the use of exclusively genetic data since transcriptomic and proteomic studies of pediatric monogenic IBD are currently very scarce. Furthermore, there are little data on how genetic loci influence age of onset in IBD. Unfortunately, due to the limited data on pediatric-specific complex IBD variants, a comparison of variants that are associated with a lower age of onset was not possible. We encourage further resequencing studies to determine what genes are most crucial in early IBD.

In summary, on the basis of our analysis, pediatric IBD and IBD-like syndromes differ from adult IBD mainly through the involvement of primary immunodeficiency in pathogenesis, as well as early changes in B-cell function. Furthermore, our comparative GO analysis according to well-established classification of pediatric IBD into five subgroups according to the age of onset (<28 days (NEO-IBD), infantile IBD with age of onset <2 years (INF-IBD), very-early-onset IBD with age of onset <6 years (VEO-IBD), early-onset IBD with age of onset <10 years (EO-IBD), and pediatric IBD with age of onset <18 years (PED-IBD)) revealed that the molecular etiology of IBD in patients with age of onset below 6 years differs most significantly from adult IBD and could be considered a separate genetic entity. As such, our results encourage frequent genetic testing for pediatric IBD patients younger than 6 years for IBD-like monogenic syndromes and support further research of the genetic architecture of pediatric IBD, especially complex pediatric IBD, which remains largely insufficiently researched. Results of our study encourage further similar systematic comprehensive studies comparing monogenetic forms and complex forms of the same disease in other diseases. This knowledge can help develop new diagnostic markers and highlights biological processes as new therapeutic targets.

## Figures and Tables

**Table 1:** Gene subgroups are defined a combination of age of onset tags. GO, Gene Ontology; N/A, not applicable; IBD inflammatory bowel disease; NEO-IBD: neonatal IBD with age of onset < 28 days; INF-IBD: infantile IBD with age of onset < 2 years; VEO-IBD: very-early-onset IBD with age of onset < 6 years; EO-IBD: early-onset IBD with age of onset < 10 years; PED-IBD: pediatric IBD with age of onset < 18 years.

Name	Subgroup Contents	Age of Onset	Number of Genes	Number of Unique Genes
ALL	Subset contains all genes with a pediatric onset tag (NEO-IBD, INF-IBD, VEO-IBD, EO-IBD and PED-IBD).	≤18 years	75	N/A
NEO	Subset contains all genes with the NEO-IBD age of onset tag.	≤28 days	19	8
INF	Subset contains all genes with the INF-IBD age of onset tag.	>28 days and ≤2 years	36	18
VEO	Subset contains all genes with the VEO-IBD age of onset tag.	>2 years and ≤6 years	37	16
EO	Subset contains all genes with the EO-IBD age of onset tag.	>6 years and ≤10 years	19	4
PED	Subset contains all genes with the PED-IBD age of onset tag.	>10 years And ≤18 years	13	0
ADULT-1	Subset contains genes based on the review of 201 IBD-associated loci.	Adult	148	145
ADULT-2	Subset contains all genes with the GO term ko05321.	Adult	49	45
UN6	Subset contains only genes with the following tags or tag combinations: NEO-IBD, NEO-IBD and INF-IBD, INF-IBD, INF-IBD and VEO-IBD	<6 years	67	N/A
OV6	Subset contains only genes with the following tags or tag combinations: VEO-IBD, VEO-IBD and EO-IBD, EO-IBD, EO-IBD and PED-IBD, PED-IBD	>6 years and ≤18 years	19	N/A

**Table 2:** Summarized Gene Ontology analysis results. All significant results of single-subset analysis are displayed in (Table S5 Supplementary Materials). INF: infantile IBD gene subset; VEO: very-early-onset IBD gene subset; EO-IBD: early-onset IBD gene subset; KEGG: Kyoto Encyclopedia of Genes and Genomes; FoxO: FOX proteins; NF-kappa B: nuclear factor kappa-light-chain-enhancer of activated B cells.

Subset	GOID ^a^	GOTerm ^b^	Term *p*-Value	Associated Gene Fraction (%)
ALL	KEGG:05340	Primary immunodeficiency	1.99 × 10^−30^	48.65
ALL	KEGG:04659	Th17 cell differentiation	3.14 × 10^−11^	11.21
ALL	KEGG:04068	FoxO signaling pathway	1.57 × 10^−7^	7.58
ALL	KEGG:04630	Jak/STAT signaling pathway	1.07 × 10^−6^	6.17
ALL	GO:0042100	B-cell proliferation	1.23 × 10^−6^	18.75
ALL	KEGG:04064	NF-kappa B signaling pathway	2.89 × 10^−6^	8.42
ALL	KEGG:04658	Th1 and Th2 cell differentiation	3.74 × 10^−5^	7.61
INF	KEGG:05340	Primary immunodeficiency	1.22 × 10^−22^	32.43
INF	KEGG:04659	Th17 cell differentiation	3.33 × 10^−6^	5.61
INF	KEGG:04658	Th1 and Th2 cell differentiation	7.36 × 10^−4^	4.35
INF	GO:0042100	B-cell proliferation	7.38 × 10^−4^	9.38
VEO	KEGG:04659	Th17 cell differentiation	8.00 × 10^−11^	8.41
VEO	KEGG:05340	Primary immunodeficiency	5.26 × 10^−9^	16.22
VEO	KEGG:04658	Th1 and Th2 cell differentiation	1.32 × 10^−6^	6.52
EO	KEGG:05340	Primary immunodeficiency	4.73 × 10^−5^	8.11
EO	KEGG:04920	Adipocytokine signaling pathway	1.55 × 10^−4^	4.35

^a^ GOID is the numerical identifier (standardized by the Gene Ontology Consortium). ^b^ GOTerm is the name of the GO term (standardized by the Gene Ontology Consortium).

**Table 3:** Summarized results of comparative Gene Ontology analysis of gene subsets. All significant results of single-subset analysis are displayed in (Table S6 Supplementary Materials) instead. UN6: gene subset containing age of onset below 6 years of age; OV6: gene subset containing age of onset above 6 years of age; ALL: gene subset containing all monogenic pediatric inflammatory bowel disease genes; ADULT-1: gene subset containing complex adult inflammatory bowel disease genes; ADULT-2: gene subset based on gene ontology term inflammatory bowel disease (IBD); KEGG: Kyoto Encyclopedia of Genes and Genomes; FoxO: FOX proteins.

Subgroups Compared	GOID ^a^	GOTerm ^b^	Term *p*-Value	Associated Gene Fraction (%)	Cluster Specific for Subset
UN6 vs. OV6	KEGG:05340	Primary immunodeficiency^-^	8.82 × 10^−32^	48.65	UN6
	KEGG:04659	Th17 cell differentiation	3.39 × 10^−12^	11.21	None
	KEGG:04660	T-cell receptor signaling pathway	1.99 × 10^−9^	9.71	OV6
	KEGG:04662	B-cell receptor signaling pathway	5.64 × 10^−8^	11.27	UN6
	KEGG:04630	Jak/STAT signaling pathway	1.57 × 10^−7^	6.17	UN6
	KEGG:04658	Th1 and Th2 cell differentiation	7.91 × 10^−6^	7.61	None
	KEGG:05321	Inflammatory bowel disease (IBD)	1.76 × 10^−5^	9.23	UN6
ALL vs. ADULT-1	KEGG:05340	Primary immunodeficiency	8.12 × 10^−22^	48.65	ALL
	KEGG:04659	Th17 cell differentiation	9.52 × 10^−19^	21.50	None
	KEGG:05321	Inflammatory bowel disease (IBD)	1.62 × 10^−16^	27.69	ADULT-1
	KEGG:04658	Th1 and Th2 cell differentiation	6.67 × 10^−10^	16.30	None
	KEGG:04630	Jak/STAT signaling pathway	3.27 × 10^−9^	11.11	ADULT-1
	KEGG:04068	FoxO signaling pathway	7.37 × 10^−5^	9.09	ALL
	GO:0042100	B cell proliferation	9.82 × 10^−4^	18.75	ALL
ALL vs. ADULT-2	KEGG:05321	Inflammatory bowel disease (IBD)	2.33 × 10^−90^	73.85	ADULT-2
	KEGG:04659	Th17 cell differentiation	3.17 × 10^−49^	33.64	ADULT-2
	KEGG:04658	Th1 and Th2 cell differentiation	2.17 × 10^−32^	28.26	ADULT-2
	KEGG:04630	Jak/STAT signaling pathway	1.72 × 10^−28^	17.28	ADULT-2
	KEGG:05340	Primary immunodeficiency	5.35 × 10^−27^	48.65	ALL
	KEGG:04068	FoxO signaling pathway	1.24 × 10^−11^	11.36	ALL
	GO:0042100	B-cell proliferation	6.79 × 10^−7^	21.88	ALL

^a^ GOID is the numerical identifier (standardized by the Gene Ontology Consortium). ^b^ GOTerm is the name of the GO term (standardized by the Gene Ontology Consortium).

## Supplementary Materials

The following are available online at https://www.mdpi.com/2075-4426/10/4/243/s1, Table S1: Review of the 201 IBD-associated loci and eQTL data; Table S2: Genes within GO term *inflammatory bowel disease*(ko05321); Table S3: Studies included for Gene Ontology analysis; Table S4: Genes causative for IBD and IBD-like syndromes; Table S5: Full results of subset Gene Ontology analysis; Table S6: Full results of comparative Gene Ontology analysis.

## References

[1] Adamiak T., Walkiewicz-Jedrzejczak D., Fish D., Brown C., Tung J., Khan K., Faubion W., Park R., Heikenen J., Yaffee M. (2013). Incidence, clinical characteristics, and natural history of pediatric IBD in Wisconsin: A population-based epidemiological study. Inflamm. Bowel Dis..

[2] Oliveira S.B., Monteiro I.M. (2017). Diagnosis and management of inflammatory bowel disease in children. BMJ.

[3] Urlep D., Blagus R., Orel R. (2015). Incidence Trends and Geographical Variability of Pediatric Inflammatory Bowel Disease in Slovenia: A Nationwide Study. Biomed. Res. Int..

[4] Van Limbergen J., Russell R.K., Drummond H.E., Aldhous M.C., Round N.K., Nimmo E.R., Smith L., Gillett P.M., McGrogan P., Weaver L.T. (2008). Definition of phenotypic characteristics of childhood-onset inflammatory bowel disease. Gastroenterology.

[5] Prenzel F., Uhlig H.H. (2009). Frequency of indeterminate colitis in children and adults with IBD—A metaanalysis. J. Crohns Colitis.

[6] Socha P., Ryzko J., Koletzko B., Celinska-Cedro D., Woynarowski M., Czubkowski P., Socha J. (2005). Essential fatty acid depletion in children with inflammatory bowel disease. Scand. J. Gastroenterol..

[7] Uhlig H.H., Schwerd T., Koletzko S., Shah N., Kammermeier J., Elkadri A., Ouahed J., Wilson D.C., Travis S.P., Turner D. (2014). The diagnostic approach to monogenic very early onset inflammatory bowel disease. Gastroenterology.

[8] Davidovics Z.H., Michail S., Nicholson M.R., Kociolek L.K., Pai N., Hansen R., Schwerd T., Maspons A., Shamir R., Szajewska H. (2019). Fecal Microbiota Transplantation for Recurrent *Clostridium difficile* Infection and Other Conditions in Children: A Joint Position Paper from the North American Society for Pediatric Gastroenterology, Hepatology, and Nutrition and the European Society for Pediatric Gastroenterology, Hepatology, and Nutrition. J. Pediatr. Gastroenterol. Nutr..

[9] De Ridder L., Assa A., Bronsky J., Romano C., Russell R.K., Afzal N.A., Hauer A.C., Knafelz D., Lionetti P., Strisciuglio C. (2019). Use of Biosimilars in Paediatric Inflammatory Bowel Disease: An Updated Position Statement of the Paediatric IBD Porto Group of ESPGHAN. J. Pediatr. Gastroenterol. Nutr..

[10] Oliva S., Thomson M., de Ridder L., Martín-de-Carpi J., Van Biervliet S., Braegger C., Dias J.A., Kolacek S., Miele E., Buderus S. (2018). Endoscopy in Pediatric Inflammatory Bowel Disease: A Position Paper on Behalf of the Porto IBD Group of the Espghan. J. Pediatr. Gastroenterol. Nutr..

[11] Thapar N., Saliakellis E., Benninga M.A., Borrelli O., Curry J., Faure C., De Giorgio R., Gupte G., Knowles C.H., Staiano A. (2018). Paediatric Intestinal Pseudo-Obstruction: Evidence and Consensus-Based Recommendations from an ESPGHAN-Led Expert Group. J. Pediatr. Gastroenterol. Nutr..

[12] Turner D., Ruemmele F.M., Orlanski-Meyer E., Griffiths A.M., Carpi J.M., Bronsky J., Veres G., Aloi M., Strisciuglio C., Braegger C.P. (2018). Management of Paediatric Ulcerative Colitis, Part 1: Ambulatory Care—An Evidence-Based Guideline from ECCO and ESPGHAN. J. Pediatr. Gastroenterol. Nutr..

[13] Turner D., Ruemmele F.M., Orlanski-Meyer E., Griffiths A.M., Carpi J.M., Bronsky J., Veres G., Aloi M., Strisciuglio C., Braegger C.P. (2018). Management of Paediatric Ulcerative Colitis, Part 2: Acute Severe Colitis; An Evidence-based Consensus Guideline from ECCO and ESPGHAN. J. Pediatr. Gastroenterol. Nutr..

[14] Lovasz B.D., Lakatos L., Horvath A., Szita I., Pandur T., Mandel M., Vegh Z., Golovics P.A., Mester G., Balogh M. (2013). Evolution of disease phenotype in adult and pediatric onset Crohn’s disease in a population-based cohort. World J. Gastroenterol..

[15] Hartono S., Ippoliti M.R., Mastroianni M., Torres R., Rider N.L. (2018). Gastrointestinal Disorders Associated with Primary Immunodeficiency Diseases. Clin. Rev. Allergy Immunol..

[16] Lega S., Pin A., Arrigo S., Cifaldi C., Girardelli M., Bianco A.M., Malamisura M., Angelino G., Faraci S., Rea F. (2020). Diagnostic Approach to Monogenic Inflammatory Bowel Disease in Clinical Practice: A Ten-Year Multicentric Experience. Inflamm. Bowel Dis..

[17] Crowley E., Warner N., Pan J., Khalouei S., Elkadri A., Fiedler K., Foong J., Turinsky A.L., Bronte-Tinkew D., Zhang S. (2020). Prevalence and Clinical Features of Inflammatory Bowel Diseases Associated with Monogenic Variants, Identified by Whole-Exome Sequencing in 1000 Children at a Single Center. Gastroenterology.

[18] Ashton J.J., Mossotto E., Stafford I.S., Haggarty R., Coelho T.A.F., Batra A., Afzal N.A., Mort M., Bunyan D., Beattie R.M. (2020). Genetic Sequencing of Pediatric Patients Identifies Mutations in Monogenic Inflammatory Bowel Disease Genes that Translate to Distinct Clinical Phenotypes. Clin. Transl. Gastroenterol..

[19] Jostins L., Ripke S., Weersma R.K., Duerr R.H., McGovern D.P., Hui K.Y., Lee J.C., Schumm L.P., Sharma Y., Anderson C.A. (2012). Host-microbe interactions have shaped the genetic architecture of inflammatory bowel disease. Nature.

[20] Kenny E.E., Pe’er I., Karban A., Ozelius L., Mitchell A.A., Ng S.M., Erazo M., Ostrer H., Abraham C., Abreu M.T. (2012). A genome-wide scan of Ashkenazi Jewish Crohn’s disease suggests novel susceptibility loci. PLoS Genet..

[21] Yamazaki K., Umeno J., Takahashi A., Hirano A., Johnson T.A., Kumasaka N., Morizono T., Hosono N., Kawaguchi T., Takazoe M. (2013). A genome-wide association study identifies 2 susceptibility Loci for Crohn’s disease in a Japanese population. Gastroenterology.

[22] Yang S.K., Hong M., Zhao W., Jung Y., Baek J., Tayebi N., Kim K.M., Ye B.D., Kim K.J., Park S.H. (2014). Genome-wide association study of Crohn’s disease in Koreans revealed three new susceptibility loci and common attributes of genetic susceptibility across ethnic populations. Gut.

[23] Liu J.Z., van Sommeren S., Huang H., Ng S.C., Alberts R., Takahashi A., Ripke S., Lee J.C., Jostins L., Shah T. (2015). Association analyses identify 38 susceptibility loci for inflammatory bowel disease and highlight shared genetic risk across populations. Nat. Genet..

[24] Ellinghaus D., Jostins L., Spain S.L., Cortes A., Bethune J., Han B., Park Y.R., Raychaudhuri S., Pouget J.G., Hübenthal M. (2016). Analysis of five chronic inflammatory diseases identifies 27 new associations and highlights disease-specific patterns at shared loci. Nat. Genet..

[25] De Lange K.M., Moutsianas L., Lee J.C., Lamb C.A., Luo Y., Kennedy N.A., Jostins L., Rice D.L., Gutierrez-Achury J., Ji S.G. (2017). Genome-wide association study implicates immune activation of multiple integrin genes in inflammatory bowel disease. Nat. Genet..

[26] Scherr R., Essers J., Hakonarson H., Kugathasan S. (2009). Genetic determinants of pediatric inflammatory bowel disease: Is age of onset genetically determined?. Dig. Dis..

[27] Ostrowski J., Paziewska A., Lazowska I., Ambrozkiewicz F., Goryca K., Kulecka M., Rawa T., Karczmarski J., Dabrowska M., Zeber-Lubecka N. (2016). Genetic architecture differences between pediatric and adult-onset inflammatory bowel diseases in the Polish population. Sci. Rep..

[28] Cleynen I., Boucher G., Jostins L., Schumm L.P., Zeissig S., Ahmad T., Andersen V., Andrews J.M., Annese V., Brand S. (2016). Inherited determinants of Crohn’s disease and ulcerative colitis phenotypes: A genetic association study. Lancet.

[29] Venkateswaran S., Prince J., Cutler D.J., Marigorta U.M., Okou D.T., Prahalad S., Mack D., Boyle B., Walters T., Griffiths A. (2018). Enhanced Contribution of HLA in Pediatric Onset Ulcerative Colitis. Inflamm. Bowel Dis..

[30] Manolio T.A., Collins F.S., Cox N.J., Goldstein D.B., Hindorff L.A., Hunter D.J., McCarthy M.I., Ramos E.M., Cardon L.R., Chakravarti A. (2009). Finding the missing heritability of complex diseases. Nature.

[31] Joshi H.J., Hansen L., Narimatsu Y., Freeze H.H., Henrissat B., Bennett E., Wandall H.H., Clausen H., Schjoldager K.T. (2018). Glycosyltransferase genes that cause monogenic congenital disorders of glycosylation are distinct from glycosyltransferase genes associated with complex diseases. Glycobiology.

[32] Batura V., Muise A.M. (2018). Very early onset IBD: Novel genetic aetiologies. Curr. Opin. Allergy Clin. Immunol..

[33] Rivas M.A., Beaudoin M., Gardet A., Stevens C., Sharma Y., Zhang C.K., Boucher G., Ripke S., Ellinghaus D., Burtt N. (2011). Deep resequencing of GWAS loci identifies independent rare variants associated with inflammatory bowel disease. Nat. Genet..

[34] Huang H., Fang M., Jostins L., UmićevićMirkov M., Boucher G., Anderson C.A., Andersen V., Cleynen I., Cortes A., Crins F. (2017). Fine-mapping inflammatory bowel disease loci to single-variant resolution. Nature.

[35] Zerbino D.R., Achuthan P., Akanni W., Amode M.R., Barrell D., Bhai J., Billis K., Cummins C., Gall A., Girón C.G. (2018). Ensembl 2018. Nucleic Acids Res..

[36] Lonsdale J., Thomas J., Salvatore M., Phillips R., Lo E., Shad S., Hasz R., Walters G., Garcia F., Young N. (2013). The Genotype-Tissue Expression (GTEx) project. Nat. Genet..

[37] Kanehisa M., Sato Y., Kawashima M., Furumichi M., Tanabe M. (2016). KEGG as a reference resource for gene and protein annotation. Nucleic Acids Res..

[38] Shannon P., Markiel A., Ozier O., Baliga N.S., Wang J.T., Ramage D., Amin N., Schwikowski B., Ideker T. (2003). Cytoscape: A software environment for integrated models of biomolecular interaction networks. Genome Res..

[39] Bindea G., Mlecnik B., Hackl H., Charoentong P., Tosolini M., Kirilovsky A., Fridman W.H., Pagès F., Trajanoski Z., Galon J. (2009). ClueGO: A Cytoscape plug-in to decipher functionally grouped gene ontology and pathway annotation networks. Bioinformatics.

[40] Suzuki T., Sasahara Y., Kikuchi A., Kakuta H., Kashiwabara T., Ishige T., Nakayama Y., Tanaka M., Hoshino A., Kanegane H. (2017). Targeted Sequencing and Immunological Analysis Reveal the Involvement of Primary Immunodeficiency Genes in Pediatric IBD: A Japanese Multicenter Study. J. Clin. Immunol..

[41] Charbit-Henrion F., Parlato M., Hanein S., Duclaux-Loras R., Nowak J., Begue B., Rakotobe S., Bruneau J., Fourrage C., Alibeu O. (2018). Diagnostic Yield of Next-Generation Sequencing in Very Early-Onset Inflammatory Bowel Diseases: A Multicenter Study. J. Crohns Colitis.

[42] Shaw K.A., Cutler D.J., Okou D., Dodd A., Aronow B.J., Haberman Y., Stevens C., Walters T.D., Griffiths A., Baldassano R.N. (2019). Genetic variants and pathways implicated in a pediatric inflammatory bowel disease cohort. Genes Immun..

[43] Wu W., Chen F., Liu Z., Cong Y. (2016). Microbiota-specific Th17 Cells: Yin and Yang in Regulation of Inflammatory Bowel Disease. Inflamm. Bowel Dis..

[44] Chaudhry A., Rudra D., Treuting P., Samstein R.M., Liang Y., Kas A., Rudensky A.Y. (2009). CD4^+^ regulatory T cells control T_H_17 responses in a Stat3-dependent manner. Science.

[45] Van Lierop P.P., Swagemakers S.M., de Bie C.I., Middendorp S., van Baarlen P., Samsom J.N., van Ijcken W.F., Escher J.C., van der Spek P.J., Nieuwenhuis E.E. (2013). Gene expression analysis of peripheral cells for subclassification of pediatric inflammatory bowel disease in remission. PLoS ONE.

[46] Hueber W., Sands B.E., Lewitzky S., Vandemeulebroecke M., Reinisch W., Higgins P.D., Wehkamp J., Feagan B.G., Yao M.D., Karczewski M. (2012). Secukinumab, a human anti-IL-17A monoclonal antibody, for moderate to severe Crohn’s disease: Unexpected results of a randomised, double-blind placebo-controlled trial. Gut.

[47] Fuss I. (2011). IL-17: Intestinal effector or protector?. Mucosal Immunol..

[48] Lee J.S., Tato C.M., Joyce-Shaikh B., Gulen M.F., Gulan F., Cayatte C., Chen Y., Blumenschein W.M., Judo M., Ayanoglu G. (2015). Interleukin-23-Independent IL-17 Production Regulates Intestinal Epithelial Permeability. Immunity.

[49] Brockmann L., Giannou A.D., Gagliani N., Huber S. (2017). Regulation of T_H_17 Cells and Associated Cytokines in Wound Healing, Tissue Regeneration, and Carcinogenesis. Int. J. Mol. Sci..

[50] Walrath T., Malizia R.A., Zhu X., Sharp S.P., D’Souza S.S., Lopez-Soler R., Parr B., Kartchner B., Lee E.C., Stain S.C. (2020). IFN-γ and IL-17A regulate intestinal crypt production of CXCL10 in the healthy and inflamed colon. Am. J. Physiol. Gastrointest. Liver Physiol..

[51] Smith M.K., Pai J., Panaccione R., Beck P., Ferraz J.G., Jijon H. (2019). Crohn’s-like disease in a patient exposed to anti-Interleukin-17 blockade (Ixekizumab) for the treatment of chronic plaque psoriasis: A case report. BMC Gastroenterol..

[52] Olivera P.A., Lasa J.S., Bonovas S., Danese S., Peyrin-Biroulet L. (2020). Safety of Janus Kinase Inhibitors in Patients with Inflammatory Bowel Diseases or Other Immune-mediated Diseases: A Systematic Review and Meta-Analysis. Gastroenterology.

[53] Salas A., Hernandez-Rocha C., Duijvestein M., Faubion W., McGovern D., Vermeire S., Vetrano S., Vande Casteele N. (2020). JAK-STAT pathway targeting for the treatment of inflammatory bowel disease. Nat. Rev. Gastroenterol. Hepatol..

