# Supplementary material for: Molecular Genetic Architecture of Monogenic Pediatric IBD Differs from Complex Pediatric and Adult IBD
Source: J Pers Med. 2020 Nov 26;10(4):243. doi: 10.3390/jpm10040243 (PMC7712254; doi:10.3390/jpm10040243)
Supplement: Supplementary file 1 [file jpm-10-00243-s001.zip › SupplementaryTableS1_ComplexIBD_LociAndEQTL.pdf]

Table S1 - Review of the 201 IBD associated loci and eQTL data.

Best reported SNP[a]	Global MAF	Variant type	Reported causal or candidate gene(s)[a]	Reported eQTL[b]	Best eQTL match[b]	Do reported causal gene and eQTL match?
rs17391694	0.029	Intergenic	N/A	FUBP1	FUBP1	No reported causal gene
rs6679677	0.0258	Upstream	DCLRE1B, PTPN22	OLFML3, LRIG2, DCLRE1B, PTPN22	PTPN22	Matching causal gene and eQTL
rs3897478	0.1927	Downstream	ADAM30, NOTCH2	NOTCH2, ADAM30	ADAM30	Matching causal gene and eQTL
rs9286879	0.4748	Intron	TNFSF18, FASLG	TNFSF18	TNFSF18	Matching causal gene and eQTL
rs1728918	0.1689	Upstream	UCN	NRBP1, UCN	UCN	Matching causal gene and eQTL
rs10865331	0.4527	Intergenic	B3GNT2	B3GNT2	B3GNT2	Matching causal gene and eQTL
rs6716753	0.1334	Intron	SP140	TRIP12, SLC16A14, SP140	SP140	Matching causal gene and eQTL
rs12994997	0.3926	Intron	INPP5D, ATG16L1	UGT1A10, RPL17P11, SCARNA5, ATG16L1	ATG16L1	Matching causal gene and eQTL
rs6837335	0.4289	Intron	TXK, TEC, SLC10A4	OCIAD1, CNGA1, NFXL1, SLC10A4	SLC10A4	Matching causal gene and eQTL
rs13126505	0.0174	Intron	NFKB1, SLC39A8, BANK1	SLC39A8	SLC39A8	Matching causal gene and eQTL
rs10065637	0.0909	Intron	IL31RA, IL6ST	ANKRD55, MIER3, IL31RA, IL6ST	IL31RA, IL6ST	Matching causal gene and eQTL
rs7702331	0.3462	Intron	N/A	FCHO2, RPL7P22	FCHO2	No reported causal gene
rs17695092	0.1224	Intron	CPEB4	CREBRF, CPEB4	CPEB4	Matching causal gene and eQTL
rs12663356	0.4687	Intergenic	N/A	N/A	N/A	No reported causal gene
rs9264942	0.3676	Intron	PSORS1C1, NFKBIL1, HLA-C, MICB	PSORS1C1, HLA-C, MICB	PSORS1C1, HLA-C, MICB	Matching causal gene and eQTL
rs9491697	0.4046	Intron	RSPO3	PRELID1P1, RSPO3	RSPO3	Matching causal gene and eQTL
rs13204742	0.0611	Regulatory region variant	N/A	KIAA0408, THEMIS	KIAA0408	No reported causal gene
rs212388	0.4491	Intron	TAGAP	TMEM181, C6orf99, TAGAP	TAGAP	Matching causal gene and eQTL

rs10486483	0.1745	Intron	N/A	CBX3, HOXA10	CBX3	No reported causal gene
rs864745	0.3027	Intron	JAZF1, CREB5	JAZF1-AS1, JAZF1, HOXA5	JAZF1	Matching causal gene and eQTL
rs7015630	0.2598	Intergenic	RIPK2	RP11-37B2.1	RP11-37B2.1	Causal gene and eQTL mismatch
rs6651252	0.1524	Intron	N/A	PVT1, MYC	PVT1	No reported causal gene
rs3764147	0.3059	Missense	LACC1	N/A	LACC1	N/A (Missense mutation)
rs16967103	0.1643	Intergenic	SPRED1, RASGRP1	RASGRP1	RASGRP1	Matching causal gene and eQTL
rs2066847	0.0060	Frameshift	NOD2	N/A	N/A	Matching causal gene and eQTL
rs2945412	0.3798	Intron	LGALS9, NOS2, KSR1	KSR1, TNFAIP1, LGALS9, NOS2	LGALS9, NOS2, KSR1	Matching causal gene and eQTL
rs2024092	0.2390	Intron	GPX4, HMHA1	GPX4	GPX4	Matching causal gene and eQTL
rs4802307	0.1338	Upstream	N/A	PPP5C	PPP5C	No reported causal gene
rs516246	0.3207	Intron	FUT2, SPHK2, IZUMO1, DBP	FUT2	FUT2	Matching causal gene and eQTL
rs2284553	0.2917	Intron	IFNGR2, IFNAR1, IFNAR2, IL10RB, GART, TMEM50B	TMEM50B, IFNGR2	TMEM50B	Matching causal gene and eQTL
rs10797432	0.4195	Downstream	FAM213B, MMEL1, TNFRSF14	FAM213B, MMEL1	FAM213B, MMEL1	Matching causal gene and eQTL
rs6426833	0.4141	Intergenic	N/A	OTUD3	OTUD3	No reported causal gene
rs2816958	0.1456	Intron	NR5A2	ASCL5, KIF21B, NR5A2, INAVA	NR5A2	Matching causal gene and eQTL
rs1016883	0.2023	Intron	RFTN2, PLCL1	RFTN2, PLCL1	RFTN2, PLCL1	Matching causal gene and eQTL
rs17229285	0.3630	Intron	N/A	MARS2	MARS2	No reported causal gene
rs9847710	0.4503	Intron	PRKCD, ITIH4	SFMBT1, ITIH4-AS1, ITHI4	ITIH4	Matching causal gene and eQTL
rs3774959	0.3361	Intron	NFKB1, MANBA	BDH2, NFKB1	NFKB1	Matching causal gene and eQTL
rs11739663	0.2406	Intergenic	SLC9A3, EXOC3	SLC9A3	SLC9A3	Matching causal gene and eQTL
rs254560	0.2782	Intron	C5orf66	C5orf66-AS1, PITX1, C5orf24	C5orf66	Matching causal gene and eQTL

rs6927022	0.3576	Non coding transcript exon variant	HLA-DQB1, HLA-DRA, HLA-DRB1, HLA-DQA1	HLA-DQA1, HLA-DRB1, HLA-DRA, HLA-DQB1	HLA-DQB1, HLA-DRA, HLA-DRB1, HLA-DQA1	Matching causal gene and eQTL
rs798502	0.2099	Intron	AMZ1, GNA12	GNA12	GNA12	Matching causal gene and eQTL
rs4722672	0.3087	Intron	N/A	HOXA13, KIAA0087	HOXA13	No reported causal gene
rs4380874	0.2798	Intergenic	SLC26A3, DLD, LAMB1	DLD, SLC26A3	SLC26A3, DLD	Matching causal gene and eQTL
rs4728142	0.2945	Upstream	TSPAN33, TNPO3, IRF5	IRF5	IRF5	Matching causal gene and eQTL
rs483905	0.2578	Intron	MAML2, JRKL, CCDC82	CCDC82	CCDC82	Matching causal gene and eQTL
rs561722	0.3914	Upstream	NXPE1, NXPE4	NXPE1	NXPE1	Matching causal gene and eQTL
rs28374715	0.1849	Intron	EXD1, OIP5, NUSAP1, RTF1, INO80, CHP1, NDUFAF1	NDUFAF1, OIP5-AS1	NDUFAF1	Matching causal gene and eQTL
rs11150589	0.4073	Upstream	ITGAL	NPIPB13, NPIPB12, SEZ6L2, ITGAL	ITGAL	Matching causal gene and eQTL
rs1728785	0.1853	Intron	ZFP90, CDH3	ZFP90	ZFP90	Matching causal gene and eQTL
rs7210086	0.1677	Downstream	N/A	N/A		No reported causal gene
rs1126510	0.2560	3-prime utr	CALM3	DACT3, PTGIR, CALM3	CALM3	Matching causal gene and eQTL
rs6088765	0.4575	Intron	UQCC1, CEP250, PROCR	FAM83C, UQCC1, EDEM2	UQCC1	Matching causal gene and eQTL
rs6017342	0.2784	Non coding transcript exon variant	C20orf62, SERINC3, PKIG, TTPAL, ADA	SGK2, WFDC5, SDC4, JPH2	SGK2	Causal gene and eQTL mismatch
rs12103	0.3193	Synonymous	TNFRSF18, TNFRSF4	ANKRD65, ATAD3C, MRPL20, TNFRSF18	TNFRSF18	Matching causal gene and eQTL
rs35675666	0.1482	5-prime utr	TNFRSF9, PARK7	PARK7	PARK7	Matching causal gene and eQTL
rs12568930	0.2322	Regulatory region variant	N/A	WNT4, NBPF3	WNT4	No reported causal gene
rs11209026	0.0228	Missense	IL23R	N/A	IL23R	N/A (Missense mutation)

rs2651244	0.2204	Upstream	N/A	PTGER3, LRRC7	PTGER3	No reported causal gene
rs4845604	0.1490	Intron	RORC	ZNF687, TCHH, CRCT1	ZNF687	Causal gene and eQTL mismatch
rs670523	0.3574	Intron	RIT1, MSTO1, UBQLN4	RIT1	RIT1	Matching causal gene and eQTL
rs4656958	0.3325	Upstream	CD244, ITLN1, CD48, SLAMF1, F11R, USF1, SLAMF7, ARHGAP30	LINC01133, IGSF8, CD244	CD244	Matching causal gene and eQTL
rs1801274	0.4417	Missense	FCGR2A	FCGR2C	FCGR2A	N/A (Missense mutation)
rs2488389	0.2334	Intron	C1orf53, DENND1B	DENND1B	DENND1B	Matching causal gene and eQTL
rs7554511	0.1368	Intron	INAVA, KIF21B, CACNA1S	MROH3P, INAVA	INAVA	Matching causal gene and eQTL
rs3024505	0.0863	Downstream	IL10	IL10	IL10	Matching causal gene and eQTL
rs6545800	0.4639	Intron	ADCY3	ADCY3	ADCY3	Matching causal gene and eQTL
rs925255	0.3425	Intron	FOSL2	FOSL2	FOSL2	Matching causal gene and eQTL
rs10495903	0.1104	Intron	ZFP36L2, THADA	DYNC2LI1, PPM1B, THADA	THADA	Matching causal gene and eQTL
rs7608910	0.2524	Intron	AHSA2, REL, KIAA1841, C2orf74, PUS2	AHSA2	AHSA2	Matching causal gene and eQTL
rs6740462	0.1777	Intron	SPRED2	ACTR2	ACTR2	Causal gene and eQTL mismatch
rs917997	0.3063	Downstream	IL18R1, IL1RL1, IL1R1, IL1RL2, IL18RAP, IL1R2	IL18RAP, IL18R1	IL18RAP, IL18R1	Matching causal gene and eQTL
rs2111485	0.3393	Intergenic	N/A	IFIH1	IFIH1	No reported causal gene
rs1517352	0.4173	Intron	STAT1, STAT4	STAT1	STAT1	Matching causal gene and eQTL
rs2382817	0.3706	5-prime utr	ARPC2, TMBIM1, CTDSP1, SLC11A1, CXCR2, CXCR1, PNKD	TMBIM1, ARPC2, PNKD	TMBIM1, ARPC2, PNKD	Matching causal gene and eQTL
rs3749171	0.1508	Missense	GPR35	N/A	GPR35	N/A (Missense mutation)
rs4256159	0.1066	Intron	N/A	SATB1-AS1	SATB1-AS1	No reported causal gene

rs3197999	0.1919	Missense	MST1	N/A	MST1	N/A (Missense mutation)
rs2472649	0.3940	Upstream	CXCL3, IL8, CXCL6, PF4V1, CXCL5, PF4, CXCL2, CXCL1	PF4, PF4V1	PF4V1	Matching causal gene and eQTL
rs7657746	0.1945	Intron	IL2, IL21	CCNA2, CETN4P	CCNA2	Causal gene and eQTL mismatch
rs2930047	0.3714	Intron	DAP	DAP	DAP	Matching causal gene and eQTL
rs11742570	0.4571	Upstream	PTGER4	PTGER4	PTGER4	Matching causal gene and eQTL
rs1363907	0.3407	Intron	ERAP2, LNPEP, ERAP1	ERAP2	ERAP2	Matching causal gene and eQTL
rs4836519	0.1961	Intergenic	N/A	N/A		No reported causal gene
rs2188962	0.1382	Intron	CSF2, IL13, IL4, IL5, IL3, IRF1, SLC22A5, ACSL6, PDLIM4, SLC22A4	SLC22A5, ACSL6	SLC22A5, ACSL6	Matching causal gene and eQTL
rs6863411	0.3337	Intron	NDFIP1	NDFIP1	NDFIP1	Matching causal gene and eQTL
rs11741861	0.1593	Intron	TNIP1, IRGM, ZNF300P1	ZNF300P1, RBM22, IRGM	IRGM, ZNF300P1	Matching causal gene and eQTL
rs6871626	0.2920	Intron	IL12B, UBLCP1	C1QTNF2, TTC1, UBLCP1	UBLCP1	Matching causal gene and eQTL
rs12654812	0.3229	Intron	DOK3, RGS14	RGS14, MXD3	RGS14	Matching causal gene and eQTL
rs17119	0.2738	Intron	N/A	N/A		No reported causal gene
rs9358372	0.4117	Intron	CDKAL1	CDKAL1, E2F3	CDKAL1	Matching causal gene and eQTL
rs1847472	0.1925	Intron	BACH2	BACH2, GABRR2	BACH2	Matching causal gene and eQTL
rs6568421	0.1512	Regulatory region variant	PRDM1, ATG5	PRDM1, ATG5	PRDM1, ATG5	Matching causal gene and eQTL
rs3851228	0.0841	Intron	KIAA1919, TRAF3IP2, SLC16A10, REV3L, FYN	TRAF3IP2-AS1	TRAF3IP2	Matching causal gene and eQTL
rs6920220	0.0944	Intron	TNFAIP3, OLIG3	MAP3K5, HEBP2	MAP3K5	Causal gene and eQTL mismatch
rs12199775	0.0443	Intron	PHACTR2	UTRN, PHACTR2	PHACTR2	Matching causal gene and eQTL

rs1819333	0.3452	Upstream	RPS6KA2, RNASET2, CCR6	RNASET2	RNASET2	Matching causal gene and eQTL
rs1456896	0.3560	Upstream	ZPBP, IKZF1, FIGNL1	FIGNL1	FIGNL1	Matching causal gene and eQTL
rs9297145	0.3437	Intergenic	KPNA7, SMURF1	SMURF1	SMURF1	Matching causal gene and eQTL
rs1734907	0.1522	Upstream	EPO	GIGYF1, EPO	EPO	Matching causal gene and eQTL
rs38904	0.4886	Intergenic	WNT2	AC002465.2	AC002465.2	Causal gene and eQTL mismatch
rs921720	0.3788	Intron	TRIB1	TATDN1, TRIB1	TRIB1	Matching causal gene and eQTL
rs1991866	0.4285	Intron	N/A	FAM49B	FAM49B	No reported causal gene
rs10758669	0.3161	Upstream	JAK2	JAK2	JAK2	Matching causal gene and eQTL
rs4743820	0.3988	Intron	AUH, NFIL3	AL158071.2, DIRAS2, LINC00484	DIRAS2	Causal gene and eQTL mismatch
rs4246905	0.2133	Missense	TNFSF15	TNFSF15	TNFSF15	N/A (Missense mutation)
rs10781499	0.3662	Synonymous	INPP5E, SDCCAG3, CARD9, PMPCA	CARD9, SEC16A, SDCCAG3,	CARD9	Matching causal gene and eQTL
rs12722515	0.1130	Intron	IL2RA, IL15RA	RPL12P28, PFKFB3, IL15RA,	IL15RA	Matching causal gene and eQTL
rs1042058	0.3528	Synonymous	MAP3K8	MTPAP, MAP3K8	MAP3K8	Matching causal gene and eQTL
rs11010067	0.3640	Downstream	CREM	CREM, PARD3	CREM	Matching causal gene and eQTL
rs2790216	0.3986	Intron	CISD1, IPMK	CISD1	CISD1	Matching causal gene and eQTL
rs10761659	0.4794	Intergenic	ADO, ZNF365	ADO	ADO	Matching causal gene and eQTL
rs2227564	0.2246	Missense	PLAU	N/A	N/A	N/A (Missense mutation)
rs1250546	0.3454	Intron	N/A	PPIF	PPIF	No reported causal gene
rs6586030	0.0649	Intron	TSPAN14, SH2D4B	NUTM2B, FAM213A, TSPAN14	TSPAN14	Matching causal gene and eQTL
rs7911264	0.4215	Intron	N/A	N/A	N/A	No reported causal gene
rs4409764	0.4700	Upstream	NKX2-3	LINC01475, AL513542.1	LINC01475	Matching causal gene and eQTL
rs907611	0.2322	Upstream	TNNI2, LSP1	LSP1	LSP1	Matching causal gene and eQTL

rs10896794	0.2015	Intron	CNTF, LPXN	MED19, CTNND1, LPXN	LPXN	Matching causal gene and eQTL
rs11230563	0.3502	Missense	CD6	CD6	CD6	N/A (Missense mutation)
rs4246215	0.3031	3-prime utr	FADS2, FADS1	FADS2, FADS1	FADS2, FADS1	Matching causal gene and eQTL
rs559928	0.1857	Intergenic	RPS6KA4, CCDC88B, TRPT1, FLRT1	AP003774.1, CCDC88B	CCDC88B	Matching causal gene and eQTL
rs2231884	0.1775	Intergenic	RELA, FOSL1, SNX32, CTSW	SNX32, CTSW	SNX32, CTSW	Matching causal gene and eQTL
rs2155219	0.4681	Upstream	LRRC32	TSKU, OMP, LRRC32	LRRC32	Matching causal gene and eQTL
rs6592362	0.4155	Intergenic	N/A	N/A		No reported causal gene
rs630923	0.0966	Upstream	CXCR5	NLRX1	NLRX1	Causal gene and eQTL mismatch
rs11612508	0.1607	Intron	BORCS5	LOH12CR2, EMP1, BORCS5	BORCS5	Matching causal gene and eQTL
rs11564258	0.0595	Intron	MUC19, LRRK2	LRRK2	LRRK2	Matching causal gene and eQTL
rs11168249	0.3980	Intron	VDR, HDAC7	HDAC7	HDAC7	Matching causal gene and eQTL
rs7134599	0.1987	Intron	IFNG, IL26, IL22	IFNG-AS1, RPL7P42, IL26	IFNG, IL26	Matching causal gene and eQTL
rs17085007	0.1835	Regulatory region variant	USP12	SNORA27, SHISA2, USP12	USP12	Matching causal gene and eQTL
rs941823	0.1759	Intron	N/A	LHFPL6	LHFPL6	No reported causal gene
rs9557195	0.1140	Intron	GPR18, GPR183, UBAC2	UBAC2	UBAC2	Matching causal gene and eQTL
rs194749	0.2408	Regulatory region variant	ZFP36L1	ZFYVE26, PPIAP6, ZFP36L1	ZFP36L1	Matching causal gene and eQTL
rs4899554	0.1300	Downstream	FOS, MLH3	AREL1, NPC2, NEK9, FOS, MLH3	FOS, MLH3	Matching causal gene and eQTL
rs8005161	0.2530	Intron	GALC, GPR65	GALC, GPR65	GALC, GPR65	Matching causal gene and eQTL
rs17293632	0.0992	Intron	SMAD3	SMAD3	SMAD3	Matching causal gene and eQTL
rs7495132	0.1466	Intron	CRTC3	FES, PLIN1, CRCT3	CRTC3	Matching causal gene and eQTL
rs529866	0.1324	Intron	LITAF, RMI2, SOCS1	RMI2	RMI2	Matching causal gene and eQTL

rs7404095	0.4209	Intron	PRKCB	PRKCB	PRKCB	Matching causal gene and eQTL
rs26528	0.3816	Intron	RABEP2, SULT1A1, SULT1A2, NUPR1, IL27, EIF3C	TUFM, SULT1A2	SULT1A2	Matching causal gene and eQTL
rs10521318	0.0469	Intron	IRF8	MTHFSD, IRF8	IRF8	Matching causal gene and eQTL
rs3091316	0.3656	Intron	CCL2, CCL11, CCL8, CCL13, CCL7, CCL1	TLK2P1, ASIC2, CCL8	CCL8	Matching causal gene and eQTL
rs12946510	0.3099	Downstream	GSDMB, ORMDL3, LRRC3C, GRB7, ZPBP2, GSDMA, IKZF3	ORMDL3, GSDMB	GSDMB, ORMDL3	Matching causal gene and eQTL
rs12942547	0.4095	Intron	STAT5B, STAT3, STAT5A	STAT3, HSBP9, TUBG2	STAT3	Matching causal gene and eQTL
rs1292053	0.4812	Missense	TUBD1	N/A	N/A	N/A (Missense mutation)
rs1893217	0.1196	Intron	PTPN2	AP005482.1, PTPN2	PTPN2	Matching causal gene and eQTL
rs7240004	0.4994	Regulatory region variant	SMAD7	C18orf32, SCARNA17	C18orf32	Causal gene and eQTL mismatch
rs727088	0.4944	3-prime utr	CD226	DOK6, CD226, SOCS6	CD226	Matching causal gene and eQTL
rs11879191	0.2370	Intron	CTD-2369P2.12, ICAM3, ZGLP1, CTD-2369P2.10, FDX1L, RAVER1, CDC37, PDE4A, KEAP1, TYK2	ICAM1, CDC37, TYK2	CDC37, TYK2	Matching causal gene and eQTL
rs17694108	0.2324	Intergenic	CEBPG	N/A	N/A	No reported eQTL
rs11672983	0.3211	Upstream	KIR2DL1, LILRB4, NLRP7, NLRP2, KIR3DL1	KIR3DL1, FCAR	KIR3DL1	Matching causal gene and eQTL
rs6142618	0.2388	Intron	TM9SF4, PLAGL2, KIF3B, ASXL1, POFUT1, C20orf112	RSL24D1P6, TSPY26P, KIF3B	KIF3B	Matching causal gene and eQTL
rs4911259	0.4255	Intron	DNMT3B	DNMT3B, COMMD7, MAPRE1	DNMT3B	Matching causal gene and eQTL
rs1569723	0.2278	Upstream	CD40, MMP9, PLTP	CD40	CD40	Matching causal gene and eQTL
rs913678	0.3950	Regulatory region variant	CEBPB	SPATA2	SPATA2	Causal gene and eQTL mismatch

rs259964	0.3806	Intron	ZNF831, CTSZ	EDN3, CDH26, CTSZ	CTSZ	Matching causal gene and eQTL
rs6062504	0.3500	Intron	LIME1, TNFRSF6B, SLC2A4RG, ZGPAT	STMN3, ARFRP1	SLC2A4RG, ZGPAT	Matching causal gene and eQTL
rs2823286	0.2346	Intron	N/A	AJ009632.2, MIR99AHG	MIR99AHG	No reported causal gene
rs2836878	0.2093	Regulatory region variant	PSMG1	AF064858.1, AF064858.3, ETS2	ETS2	Causal gene and eQTL mismatch
rs7282490	0.4095	Intron	ICOSLG	ICOSLG	ICOSLG	Matching causal gene and eQTL
rs2266959	0.2272	Intron	YDJC, SDF2L1, UBE2L3, CCDC116	CCDC116	CCDC116	Matching causal gene and eQTL
rs2412970	0.4421	Intron	LIF, MTMR3, OSM	MTMR3	MTMR3	Matching causal gene and eQTL
rs2413583	0.1691	Intron	SYNGR1, RPL3, ATF4, TAB1, APOBEC3G	TMEM184B, APOBEC3C, RPL3	RPL3	Matching causal gene and eQTL
rs1748195	0.4355	Intron	ANGPTL3, AL138847.1, DOCK7, USP1	DOCK7, USP1	DOCK7, USP1	Matching causal gene and eQTL
rs34856868	0.0082	Missense	BTBD8	N/A	N/A	N/A (Missense mutation)
rs11583043	0.2360	Intron	SLC30A, S1PR1	AC093157.1, SLC30A7	SLC30A7	Causal gene and eQTL mismatch
rs6025	0.0060	Missense	F5	N/A	N/A	N/A (Missense mutation)
rs10798069	0.3253	Intron	PTGS2, PLA2G4A	PLA2G4A, ODR4	PLA2G4A	Matching causal gene and eQTL
rs7555082	0.0625	Intergenic	PTPRC	N/A	N/A	No reported eQTL
rs11681525	0.0308	Intron	N/A	TEX41	TEX41	No reported causal gene
rs4664304	0.2602	3-prime utr	MARCH7, LY75, PLA2R1	LY75	LY75	Matching causal gene and eQTL
rs3116494	0.1747	Intron	ICOS, CD28, CTLA4	CD28	CD28	Matching causal gene and eQTL
rs111781203	N/A	Intergenic	N/A	N/A	N/A	No reported causal gene
rs35320439	0.4888	Intron	PDCD1, ATG4B	D2HGDH, GAL3ST2	D2HGDH	Causal gene and eQTL mismatch

rs113010081	0.0284	Downstream	FLJ78302, LTF, CCR1, CCR2, CCR3, CCR5	CCR3, SLC6A20, SACM1L	CCR3	Matching causal gene and eQTL
rs616597	0.1977	Intron	NFKBIZ	CEP97, NFKBIZ	NFKBIZ	Matching causal gene and eQTL
rs724016	0.4786	5-prime utr	ZBTB38	ZBTB38	ZBTB38	Matching causal gene and eQTL
rs2073505	0.1098	Synonymous	HGFAC	DOK7, HGFAC	HGFAC	Matching causal gene and eQTL
rs4692386	0.3888	Intergenic	N/A	N/A	N/A	No reported causal gene
rs6856616	0.2226	Intergenic	KLF3, TBC1D1	N/A	N/A	No reported eQTL
rs2189234	0.4056	Intron	N/A	PPA2	PPA2	No reported causal gene
rs395157	0.4479	Intron	OSMR, FYB, LIFR	LIFR	LIFR	Matching causal gene and eQTL
rs4703855	0.3005	Intergenic	N/A	N/A	N/A	No reported causal gene
rs564349	0.2620	3-prime utr	C5orf4, DUSP1, ERGIC1	CDC42P5, ERGIC1, DUSP1	DUSP1, ERGIC1	Matching causal gene and eQTL
rs7773324	0.3478	Intergenic	IRF4, DUSP22	N/A	N/A	No reported eQTL
rs13204048	0.3141	Intron	N/A	TUBB2BP1	TUBB2BP1	No reported causal gene
rs7758080	0.3421	Intron	TAB2	UST, PPP1R14C, TAB2	TAB2	Matching causal gene and eQTL
rs1077773	0.4880	Intron	AHR, AC019117.3	AGR3, BZW2, SNX13	AGR3	Causal gene and eQTL mismatch
rs2538470	0.3331	Intergenic	CNTNAP2	CUL1	CUL1	Causal gene and eQTL mismatch
rs17057051	0.2993	Intron	PTK2B, TRIM35, EPHX2	PTK2B, DPYSL2	PTK2B	Matching causal gene and eQTL
rs7011507	0.1102	Intergenic	N/A	N/A	N/A	No reported causal gene
rs3740415	0.4105	3-prime utr	NFKB2, TRIM8, MFSD13A	MFSD13A, SFXN2, MFSD13A	MFSD13A	Matching causal gene and eQTL
rs7954567	0.1923	Intron	CD27, TNFRSF1A, LTBR	LTBR	LTBR	Matching causal gene and eQTL
rs653178	0.1474	Intron	SH2B3, ALDH2, ATXN2	MAPKAPK5, TMEM116, SH2B3	SH2B3	Matching causal gene and eQTL
rs11064881	0.0327	Intron	PRKAB1	PRKAB1	PRKAB1	Matching causal gene and eQTL

rs9525625	0.3766	Intron	AKAP1, TFSF11	AKAP11, EPSTI1	AKAP11	Causal gene and eQTL mismatch
rs3853824	0.3309	Intron	C17orf67, DGKE	DGKE	DGKE	Matching causal gene and eQTL
rs17736589	0.0998	Intron	N/A	TIMP2, TNRC6C-AS1	TIMP2	No reported causal gene
rs9319943	0.2528	Intergenic	SEC11C, GRP	N/A	N/A	No reported eQTL
rs7236492	0.0623	Intron	NFATC1, TST	NFATC1, RBFA, SLC25A6P4	NFATC1	Matching causal gene and eQTL
rs727563	0.4690	Intron	TEF, NHP2L1, PMM1, L3MBTL2, CHADL	TEF, MEI1, POLR3H	TEF	Matching causal gene and eQTL
rs34687326	0,0307	Missense	SLAMF8	N/A	N/A	N/A (Missense mutation)
rs59043219	0,4037	Intron	IRF6	IRF6	IRF6	Matching causal gene and eQTL
rs6740847	0,4590	Upstream	ITGA4	ITGA4	ITGA4	Matching causal gene and eQTL
rs144344067	0,05	Intergenic	N/A	N/A	N/A	No reported eQTL
rs1811711	0,1533	Intergenic	CCL20	CCL20	CCL20	Matching causal gene and eQTL
rs76527535	0,1461	Intron	BOK	BOK	BOK	Matching causal gene and eQTL
rs2581828	0,4806	Downstream	RTF1	RTF1	RTF1	Matching causal gene and eQTL
rs2593855	0,3823	Intron	FOXP1	N/A	N/A	No reported eQTL
rs503734	0,3604	Intron	IMPG2	IMPG2	IMPG2	Matching causal gene and eQTL
rs56116661	0,2222	Intron	LPP	N/A	N/A	No reported eQTL
rs11734570	0,4582	Intergenic	N/A	N/A	N/A	No reported eQTL
rs17656349	0,4388	Intron	CAMK2A	CAMK2A	CAMK2A	Matching causal gene and eQTL
rs113986290	0,0331	Intron	LNC-LBCS	RP4-625H18.2	N/A	Causal gene and eQTL mismatch
rs67289879	0,0804	Intron	CCND3	MED20	N/A	Causal gene and eQTL mismatch
rs11768365	0,2649	Intron	GRID2IP	DAGLB, KDELR2	N/A	Causal gene and eQTL mismatch

rs149169037	0,0285	Downstream	ITGB8	ITGB8	ITGB8	Matching causal gene and eQTL
rs243505	0,4656	Intron	CUL1	CUL1	CUL1	Matching causal gene and eQTL
rs7911117	0,1875	Intergenic	N/A	N/A	N/A	No reported eQTL
rs111456533	0,2048	Intergenic	EEF1AKMT2	METTL10	N/A	Causal gene and eQTL mismatch
rs80244186	0,1206	Downstream	AKAP11	AKAP11	AKAP11	Matching causal gene and eQTL
rs11548656	0,0127	Missense	PLCG2	N/A	N/A	N/A (Missense mutation)
rs10492862	0,2645	Intron	CDH13	N/A	N/A	No reported eQTL
rs4256018	0,3594	Intron	FERMT1	N/A	N/A	No reported eQTL
rs138788	0,4257	Intron	TOM1	TOM1	TOM1	Matching causal gene and eQTL
rs4821544	0,3528	Intron	NCF4	NCF4	NCF4	Matching causal gene and eQTL
rs1487630	0,3528	Intergenic	N/A	N/A	N/A	No reported eQTL
rs7329174	0,2058	Intron	SLC25A15, ELF1, WBP4	ELF1	ELF1	Matching causal gene and eQTL
rs7705924	0,1072	Intron	RPL7	RPL7	RPL7	Matching causal gene and eQTL
rs10734105	0,0545	Intergenic	CPAMD8	CPAMD8	CPAMD8	Matching causal gene and eQTL
rs12677663	0,3019	Upstream	PRG2	SBSPON	N/A	No reported eQTL
rs11229030	0,2725	Upstream	PRG3	PRG3	PRG3	Matching causal gene and eQTL
rs6908425	0,3875	Intron	CDKAL1	CDKAL1	CDKAL1	Matching causal gene and eQTL
rs67025039	0,2182	Intron	FGFR1OP	FGFR1OP	FGFR1OP	Matching causal gene and eQTL
rs4986790	0,0599	Missense	TLR4	TLR4	TLR4	N/A (Missense mutation)
rs2236379	0,3654	Missense	PRKCQ	N/A	N/A	N/A (Missense mutation)
rs11221332	0,1501	Intron	EST1	EST1	EST1	Matching causal gene and eQTL
rs45450798	0,1196	Intron	PTPN2	PTPN2	PTPN2	Matching causal gene and eQTL

rs11235667	0,0215	Intergenic	N/A	N/A	N/A	No reported eQTL
rs11195128	0,2038	Intergenic	N/A	N/A	N/A	No reported eQTL

[a]Best SNP and causal or candidate genes reported by GWAS and post-GWAS. [13, 14]

[b]eQTL and best eQTL traits of loci described by fine-mapping study or Ensembl genome browser.
