# Supplementary material for: Molecular Genetic Architecture of Monogenic Pediatric IBD Differs from Complex Pediatric and Adult IBD
Source: J Pers Med. 2020 Nov 26;10(4):243. doi: 10.3390/jpm10040243 (PMC7712254; doi:10.3390/jpm10040243)
Supplement: Supplementary file 1 [file jpm-10-00243-s001.zip › SupplementaryTableS2_IBD_GeneTerms.pdf]

Table S2 - Genes within GO term Inflammatory Bowel Disease (ko05321).

KEGG GO ID[a]	Definition	Gene
K02580	NFKB1; nuclear factor NF-kappa-B p105 subunit	NFKB1
K03156	TNF, TNFA; tumor necrosis factor superfamily, member 2	TNF
K04383	IL1A; interleukin 1 alpha	IL1A
K04446	NFATC1, NFAT2, NFATC; nuclear factor of activated T-cells, cytoplasmic 1	NFATC1
K04448	JUN; transcription factor AP-1	JUN
K04500	SMAD2_3; mothers against decapentaplegic homolog 2/3	SMAD2
K04519	IL1B; interleukin 1 beta	IL1B
K04687	IFNG; interferon gamma	IFNG
K04692	STAT3; signal transducer and activator of transcription 3	STAT3
K04735	RELA; transcription factor p65	RELA
K05063	IL12RB1, CD212; interleukin 12 receptor beta-1	IL12RB1
K05064	IL12RB2; interleukin 12 receptor beta-2	IL12RB2
K05065	IL23R; interleukin 23 receptor	IL23R
K05070	IL2RG, CD132; interleukin 2 receptor gamma	IL2RG
K05071	IL4R, CD124; interleukin 4 receptor	IL4R
K05075	IL21R, CD360; interleukin 21 receptor	IL21R
K05132	IFNGR1, CD119; interferon gamma receptor 1	IFNGR1
K05133	IFNGR2; interferon gamma receptor 2	IFNGR2
K05173	IL18R1, IL1RRP, CD218a; interleukin 18 receptor 1	IL18R1
K05174	IL18RAP, ACPL, CD218b; interleukin 18 receptor accessory protein	IL18RAP
K05405	IL6; interleukin 6	IL6
K05406	IL12A; interleukin 12A	IL12A
K05425	IL12B; interleukin 12B	IL12B
K05426	IL23A; interleukin 23, alpha subunit p19	IL23A
K05428	IL5; interleukin 5	IL5
K05429	IL2; interleukin 2	IL2
K05430	IL4; interleukin 4	IL4R
K05434	IL21; interleukin 21	IL21R
K05435	IL13; interleukin 13	IL13
K05443	IL10, CSIF; interleukin 10	IL10
K05445	IL22, IL-TIF; interleukin 22	IL22

K05482	IL18, IL1F4; interleukin 18	IL18
K05489	IL17A, CTLA8; interleukin 17A	IL17A
K05494	IL17F, ML1; interleukin 17F	IL17F
K08532	NR1F1, RORA; RAR-related orphan receptor alpha	RORA
K08534	NR1F3, RORC; RAR-related orphan receptor gamma	NR1F3
K09035	CMAF, MAF; transcription factor Maf	MAF
K10159	TLR2, CD282; toll-like receptor 2	TLR2
K10160	TLR4, CD284; toll-like receptor 4	TLR4
K10163	FOXP3, IPEX; forkhead box P3	FOXP3
K10165	NOD2, CARD15; nucleotide-binding oligomerization domain-containing protein 2	NOD2
K10166	TBX21; T-box protein 21	TBX21
K10168	TLR5; toll-like receptor 5	TLR5
K11220	STAT1; signal transducer and activator of transcription 1	STAT1
K11222	STAT4; signal transducer and activator of transcription 4	STAT4
K11225	STAT6; signal transducer and activator of transcription 6	STAT6
K13375	TGFB1; transforming growth factor beta-1	TGFB1
K13376	TGFB2; transforming growth factor beta-2	TGFB2
K13377	TGFB3; transforming growth factor beta-3	TGFB3

[a]KEGG GO ID is the numerical ID of the gene defined by KEGG.
