# Supplementary material for: Molecular Genetic Architecture of Monogenic Pediatric IBD Differs from Complex Pediatric and Adult IBD
Source: J Pers Med. 2020 Nov 26;10(4):243. doi: 10.3390/jpm10040243 (PMC7712254; doi:10.3390/jpm10040243)
Supplement: Supplementary file 1 [file jpm-10-00243-s001.zip › SupplementaryTableS3_IncludedStudies.pdf]

Table S3 - Studies included for gene ontology analysis. Final list of studies where monogenic pediatric IBD and IBD-like syndrome causing genes were extracted from.

Nr.	Study title and reference	Number of analyzed patients and families
1.	Brigida I, Zoccolillo M, Cicalese MP, Pfajfer L, Barzaghi F, Scala S, et al. T-cell defects in patients with. Blood. 2018 11;132(22):2362-74.	2 patients
2.	Arnold DE, Heimall JR. A Review of Chronic Granulomatous Disease. Adv Ther. 2017 12;34(12):2543-57.	*review*
3.	Janecke AR, Heinz-Erian P, Yin J, Petersen BS, Franke A, Lechner S, et al. Reduced sodium/proton exchanger NHE3 activity causes congenital sodium diarrhea. Hum Mol Genet. 2015 Dec;24(23):6614-23.	18 patients and 16 families
4.	Zhou Q, Wang H, Schwartz DM, Stoffels M, Park YH, Zhang Y, et al. Loss-of-function mutations in TNFAIP3 leading to A20 haploinsufficiency cause an early-onset autoinflammatory disease. Nat Genet. 2016 01;48(1):67-73.	5 families
5.	Sogkas G, Dubrowinskaja N, Bergmann AK, Lentes J, Ripperger T, Fedchenko M, et al. Progressive Immunodeficiency with Gradual Depletion of B and $CD4^+$ T Cells in Immunodeficiency, Centromeric Instability and Facial Anomalies Syndrome 2 (ICF2). Diseases. 2019 Apr;7(2).	1 patient
6.	Chiriaco M, Di Matteo G, Conti F, Petricone D, De Luca M, Di Cesare S, et al. First Case of Patient With Two Homozygous Mutations in. Front Immunol. 2019;10:130.	1 patient
7.	Lucas CL, Kuehn HS, Zhao F, Niemela JE, Deenick EK, Palendira U, et al. Dominant-activating germline mutations in the gene encoding the PI(3)K catalytic subunit p110δ result in T cell senescence and human immunodeficiency. Nat Immunol. 2014 Jan;15(1):88-97.	14 patients
8.	Uhlig HH, Schwerd T, Koletzko S, Shah N, Kammermeier J, Elkadri A, et al. The diagnostic approach to monogenic very early onset inflammatory bowel disease. Gastroenterology. 2014 Nov;147(5):990-1007.e3.	1605 patients
9.	Bianco AM, Girardelli M, Tommasini A. Genetics of inflammatory bowel disease from multifactorial to monogenic forms. World J Gastroenterol. 2015 Nov 21;21(43):12296-310.	*review*
10.	Xiao Y, Wang XQ, Yu Y, Guo Y, Xu X, Gong L, et al. Comprehensive mutation screening for 10 genes in Chinese patients suffering very early onset inflammatory bowel disease. World J Gastroenterol. 2016 Jun;22(24):5578-88.	13 patients
11.	Li Q, Lee CH, Peters LA, Mastropaolo LA, Thoeni C, Elkadri A, et al. Variants in TRIM22 That Affect NOD2 Signaling Are Associated With Very-Early-Onset Inflammatory Bowel Disease. Gastroenterology. 2016 05;150(5):1196-207.	150 patients
12.	Bianco AM, Zanin V, Girardelli M, Magnolato A, Martelossi S, Martellossi S, et al. A common genetic background could explain early-onset Crohn's disease. Med Hypotheses. 2012 Apr;78(4):520-2.	*review*
13.	Avitzur Y, Guo C, Mastropaolo LA, Bahrami E, Chen H, Zhao Z, et al. Mutations in tetratricopeptide repeat domain 7A result in a severe form of very early onset inflammatory bowel disease. Gastroenterology. 2014 Apr;146(4):1028-39.	41 patients

14.	Kammermeier J, Drury S, James CT, Dziubak R, Ocaka L, Elawad M, et al. Targeted gene panel sequencing in children with very early onset inflammatory bowel disease--evaluation and prospective analysis. J Med Genet. 2014 Nov;51(11):748-55.	45 patients
15.	Salzer E, Kansu A, Sic H, Májek P, Ikincioğullari A, Dogu FE, et al. Early-onset inflammatory bowel disease and common variable immunodeficiency-like disease caused by IL-21 deficiency. J Allergy Clin Immunol. 2014 Jun;133(6):1651-9.e12.	3 patients
16.	Muise AM, Xu W, Guo CH, Walters TD, Wolters VM, Fattouh R, et al. NADPH oxidase complex and IBD candidate gene studies: identification of a rare variant in NCF2 that results in reduced binding to RAC2. Gut. 2012 Jul;61(7):1028-35.	2049 patients
17.	Wang XC. [Clinical features of X-linked agammaglobulinemia: analysis of 8 cases]. Zhonghua Er Ke Za Zhi. 2004 Aug;42(8):564-7.	8 patients
18.	Conley ME, Dobbs AK, Quintana AM, Bosompem A, Wang YD, Coustan-Smith E, et al. Agammaglobulinemia and absent B lineage cells in a patient lacking the p85α subunit of PI3K. J Exp Med. 2012 Mar;209(3):463-70.	1 patient
19.	Ferrari S, Lougaris V, Caraffi S, Zuntini R, Yang J, Soresina A, et al. Mutations of the Igbeta gene cause agammaglobulinemia in man. J Exp Med. 2007 Sep;204(9):2047-51.	1 patient
20.	Betterle C, Greggio NA, Volpato M. Clinical review 93: Autoimmune polyglandular syndrome type 1. J Clin Endocrinol Metab. 1998 Apr;83(4):1049-55.	*review*
21.	Kelsen JR, Baldassano RN, Artis D, Sonnenberg GF. Maintaining intestinal health: the genetics and immunology of very early onset inflammatory bowel disease. Cell Mol Gastroenterol Hepatol. 2015 Sep;1(5):462-76.	*review*
22.	Lübbehusen J, Thiel C, Rind N, Ungar D, Prinsen BH, de Koning TJ, et al. Fatal outcome due to deficiency of subunit 6 of the conserved oligomeric Golgi complex leading to a new type of congenital disorders of glycosylation. Hum Mol Genet. 2010 Sep;19(18):3623-33.	1 patient
23.	Huybrechts S, De Laet C, Bontems P, Rooze S, Souayah H, Sznajer Y, et al. Deficiency of Subunit 6 of the Conserved Oligomeric Golgi Complex (COG6-CDG): Second Patient, Different Phenotype. JIMD Rep. 2012;4:103-8.	1 patient
24.	Kanegane H, Agematsu K, Futatani T, Sira MM, Suga K, Sekiguchi T, et al. Novel mutations in a Japanese patient with CD19 deficiency. Genes Immun. 2007 Dec;8(8):663-70.	1 patient
25.	van Zelm MC, Smet J, van der Burg M, Ferster A, Le PQ, Schandené L, et al. Antibody deficiency due to a missense mutation in CD19 demonstrates the importance of the conserved tryptophan 41 in immunoglobulin superfamily domain formation. Hum Mol Genet. 2011 May;20(9):1854-63.	1 patient
26.	Kuijpers TW, Bende RJ, Baars PA, Grummels A, Derks IA, Dolman KM, et al. CD20 deficiency in humans results in impaired T cell-independent antibody responses. J Clin Invest. 2010 Jan;120(1):214-22.	1 patient
27.	van Zelm MC, Smet J, Adams B, Mascart F, Schandené L, Janssen F, et al. CD81 gene defect in humans disrupts CD19 complex formation and leads to antibody deficiency. J Clin Invest. 2010 Apr;120(4):1265-74.	1 patient
28.	Trotta L, Hautala T, Hämäläinen S, Syrjänen J, Viskari H, Almusa H, et al.	6 patients

	Enrichment of rare variants in population isolates: single AICDA mutation responsible for hyper-IgM syndrome type 2 in Finland. Eur J Hum Genet. 2016 10;24(10):1473-8.	
29.	Lohr NJ, Molleston JP, Strauss KA, Torres-Martinez W, Sherman EA, Squires RH, et al. Human ITCH E3 ubiquitin ligase deficiency causes syndromic multisystem autoimmune disease. Am J Hum Genet. 2010 Mar;86(3):447-53.	10 patients
30.	Christiano AM, McGrath JA, Tan KC, Uitto J. Glycine substitutions in the triple-helical region of type VII collagen result in a spectrum of dystrophic epidermolysis bullosa phenotypes and patterns of inheritance. Am J Hum Genet. 1996 Apr;58(4):671-81.	6 families
31.	Kelsen JR, Dawany N, Moran CJ, Petersen BS, Sarmady M, Sasson A, et al. Exome sequencing analysis reveals variants in primary immunodeficiency genes in patients with very early onset inflammatory bowel disease. Gastroenterology. 2015 Nov;149(6):1415-24.	65 patients
32.	Kobayashi K, Fujita K, Okino F, Kajii T. An abnormality of neutrophil adhesion: autosomal recessive inheritance associated with missing neutrophil glycoproteins. Pediatrics. 1984 May;73(5):606-10.	1 patient
33.	Kelsen JR, Dawany N, Martinez A, Grochowski CM, Maurer K, Rappaport E, et al. A de novo whole gene deletion of XIAP detected by exome sequencing analysis in very early onset inflammatory bowel disease: a case report. BMC Gastroenterol. 2015 Nov;15:160.	1 patient
34.	Weinstein EJ, Bourner M, Head R, Zakeri H, Bauer C, Mazzarella R. URP1: a member of a novel family of PH and FERM domain-containing membrane-associated proteins is significantly over-expressed in lung and colon carcinomas. Biochim Biophys Acta. 2003 Apr;1637(3):207-16.	40 patients
35.	Sunseri WM, Kugathasan S, Keljo DJ, Greer JB, Ranganathan S, Cross RK, et al. IBD LIVE Case Series--Case 3: Very Early-Onset Inflammatory Bowel Disease: When Genetic Testing Proves Beneficial. Inflamm Bowel Dis. 2015 Dec;21(12):2958-68.	*review*
36.	Schubert D, Bode C, Kenefeck R, Hou TZ, Wing JB, Kennedy A, et al. Autosomal dominant immune dysregulation syndrome in humans with CTLA4 mutations. Nat Med. 2014 Dec;20(12):1410-16.	19 patients
