# Supplementary material for: Molecular Genetic Architecture of Monogenic Pediatric IBD Differs from Complex Pediatric and Adult IBD
Source: J Pers Med. 2020 Nov 26;10(4):243. doi: 10.3390/jpm10040243 (PMC7712254; doi:10.3390/jpm10040243)
Supplement: Supplementary file 1 [file jpm-10-00243-s001.zip › SupplementaryTableS4_GeneList.pdf]

Table S4 – Genes causative for IBD and IBD-like syndromes. Syndromes with an age of onset range spanning multiple groups received all applicable gene onset subgroup tags.

Gene	Associated syndrome and reference	Age of onset category (tag)
ADAM17	ADAM17 deficiency (1)	NEO-IBD
IL10	NEO-IBD due to IL-10 signaling defects (1, 2)	NEO-IBD
IL10RA	NEO-IBD due to IL-10 signaling defects (1-3)	NEO-IBD
IL10RB	NEO-IBD due to IL-10 signaling defects (1-3)	NEO-IBD
TRIM22	Reported as causative in NEO-IBD patient (4)	NEO-IBD
EGFR	Neonatal inflammatory skin and bowel disease 2 (2, 5)	NEO-IBD
TTC7A	TTC7A deficiency (1, 6)	NEO-IBD
EPCAM	Tufting enteropathy (7)	NEO-IBD
WAS	B- and T-cell development WAS (1)	NEO-IBD, INF-IBD
DKC1	Hoyeraal–Hreidarsson syndrome (1)	NEO-IBD, INF-IBD
RTEL1	Hoyeraal–Hreidarsson syndrome (1)	NEO-IBD, INF-IBD
IL21	IL-21 deficiency (8)	NEO-IBD, INF-IBD
NCF4	Reported as causative in NEO-IBD and INF-IBD patients (9)	NEO-IBD, INF-IBD
ARPC1B	WAS-like combined immunodeficiency (10)	NEO-IBD, INF-IBD
CYBA	Chronic granulomatous disease subtype (11)	NEO-IBD, INF-IBD
CYBB	Chronic granulomatous disease subtype (11)	NEO-IBD, INF-IBD
NCF1	Chronic granulomatous disease subtype (11)	NEO-IBD, INF-IBD
NCF2	Chronic granulomatous disease subtype (11)	NEO-IBD, INF-IBD
BTK	Agammaglobulinemia or Hypogammaglobulinemia (1, 12)	INF-IBD
PIK3R1	Agammaglobulinemia or Hypogammaglobulinemia (1, 13)	INF-IBD
CD79B	Agammaglobulinemia or Hypogammaglobulinemia (14)	INF-IBD
AIRE	Autoimmune Polyendocrinopathy Candidiasis Ectodermal Dystrophy (2, 5, 15)	INF-IBD
CHD7	CHARGE syndrome (16)	INF-IBD
COG6	Congenital disorder of glycosylation, type III (2, 5, 17, 18)	INF-IBD
CD19	Common variable immune deficiency 3 (16, 19, 20)	INF-IBD
MS4A1	Common variable immune deficiency 5 (16, 21)	INF-IBD
CD81	Common variable immune deficiency 6 (16, 22)	INF-IBD
MEFV	Familial Mediterranean fever (1)	INF-IBD
CD40LG	Hyper IgM syndrome type 1 (1)	INF-IBD
AICDA	Hyper IgM syndrome type 2 (1, 23)	INF-IBD
CD40	Hyper IgM syndrome type 3 (16)	INF-IBD
FOXP3	IPEX syndrome (1)	NEO-IBD, INF-IBD, VEO-IBD
DCLRE1C	Omenn syndrome (16)	INF-IBD
IL7R	Omenn syndrome (16)	INF-IBD
RAG1	Omenn syndrome (16)	INF-IBD
RAG2	Omenn syndrome (16)	INF-IBD
PLCG2	Phospholipase C-γ2 defects (1)	INF-IBD

ZBTB24	Immunodeficiency, Centromeric Instability and Facial Anomalies Syndrome 2 (24)	INF-IBD
CARD9	Hereditary candidasis (25)	INF-IBD
PIK3CD	Immunodeficiency 14 (26)	INF-IBD
ITCH	Autoimmune disease, multisystem with facial dysmorphism (2, 5, 27)	INF-IBD, VEO-IBD
COL7A1	Dystrophic bullosa (1, 28)	INF-IBD, VEO-IBD
IL2RA	Immunodeficiency 41 with lymphoproliferation and autoimmunity (1)	INF-IBD, VEO-IBD
STAT1	Immunodeficiency 31a, 31b and 31c (1)	INF-IBD, VEO-IBD
ADA	Severe combined immunodeficiency (1)	INF-IBD, VEO-IBD
CD3G	Severe combined immunodeficiency (1)	INF-IBD, VEO-IBD
IL2RG	Severe combined immunodeficiency (1)	INF-IBD, VEO-IBD
LIG4	Severe combined immunodeficiency (1)	INF-IBD, VEO-IBD
CASP8	Caspase 8 deficiency (2, 5)	VEO-IBD
CR2	Common variable immune deficiency 7 (16)	VEO-IBD
LRBA	Common variable immune deficiency 8 (16)	VEO-IBD
TGFBR1	Loeys-Dietz syndrome, type 1 (2, 5)	VEO-IBD
TGFBR2	Loeys-Dietz syndrome, type 2 (2, 5)	VEO-IBD
MASP2	MASP2 deficiency (2, 5)	VEO-IBD
ITK	Severe combined immunodeficiency (16)	VEO-IBD
LCK	Severe combined immunodeficiency (16)	VEO-IBD
ZAP70	Severe combined immunodeficiency (16)	VEO-IBD
TNFRSF13B	TACI deficiency (2, 5, 16)	VEO-IBD
SKIV2L	Trichohepatoenteric syndrome (1)	INF-IBD, VEO-IBD, EO-IBD
TTC37	Trichohepatoenteric syndrome (1)	VEO-IBD
MSH5	Reported as causative in VEO-IBD patient (29)	VEO-IBD
SH2D1A	X-linked lymphoproliferative syndrome 1 (1)	VEO-IBD
XLP1	X-linked lymphoproliferative syndrome 1 (16)	VEO-IBD
SLAMF1	X-linked lymphoproliferative syndrome 1 with defective SLAMF1 (16)	VEO-IBD
XLP2	X-linked lymphoproliferative syndrome 2 (16)	VEO-IBD
UNG	Hyper IgM syndrome type 5 (16)	VEO-IBD, EO-IBD
SLC9A3	Congenital sodium diarrhea (30)	VEO-IBD, EO-IBD
ITGB2	Leukocyte adhesion deficiency 1 (1, 31)	INF-IBD, VEO-IBD, EO-IBD, PED-IBD
XIAP	X-linked lymphoproliferative syndrome 2 (1, 32)	INF-IBD, VEO-IBD, EO-IBD, PED-IBD
G6PC3	Congenital neutropenia (1)	VEO-IBD, EO-IBD, PED-IBD
GUCY2C	Familial diarrhea (1)	VEO-IBD, EO-IBD, PED-IBD

SLC37A4	Glycogen storage disease type lb (1)	VEO-IBD, EO-IBD, PED-IBD
HPS1	Hermansky-Pudlak syndrome 1 (1)	VEO-IBD, EO-IBD, PED-IBD
HPS4	Hermansky-Pudlak syndrome 4 (1)	VEO-IBD, EO-IBD, PED-IBD
HPS6	Hermansky-Pudlak syndrome 6 (1)	VEO-IBD, EO-IBD, PED-IBD
DOCK8	Hyper IgE syndrome (1)	EO-IBD
FERMT1	Kindler syndrome (1, 33)	VEO-IBD, EO-IBD, PED-IBD
MVK	Mevalonate kinase deficiency (29)	EO-IBD
TNFAIP3	Early-onset autoinflammatory disease (34)	EO-IBD
PTEN	PTEN hamartoma tumor syndrome (35)	EO-IBD
IKBKG	X-linked ectodermal immunodeficiency (1)	EO-IBD
CTLA4	Autoimmune lymphoproliferative syndrome type V (2, 5, 36)	EO-IBD, PED-IBD
ICOS	Common variable immune deficiency 1 (1)	EO-IBD, PED-IBD
STXBP2	Familial hemophagocytic lymphohistiocytosis (1) type 5	EO-IBD, PED-IBD
STAT3	Hyper IgE syndrome (29)	EO-IBD, PED-IBD

Literature

1. Uhlig HH, Schwerd T, Koletzko S, Shah N, Kammermeier J, Elkadri A, et al. The diagnostic approach to monogenic very early onset inflammatory bowel disease. Gastroenterology. 2014;147(5):990-1007.e3.
2. Bianco AM, Girardelli M, Tommasini A. Genetics of inflammatory bowel disease from multifactorial to monogenic forms. World J Gastroenterol. 2015;21(43):12296-310.
3. Xiao Y, Wang XQ, Yu Y, Guo Y, Xu X, Gong L, et al. Comprehensive mutation screening for 10 genes in Chinese patients suffering very early onset inflammatory bowel disease. World J Gastroenterol. 2016;22(24):5578-88.
4. Li Q, Lee CH, Peters LA, Mastropaolo LA, Thoeni C, Elkadri A, et al. Variants in TRIM22 That Affect NOD2 Signaling Are Associated With Very-Early-Onset Inflammatory Bowel Disease. Gastroenterology. 2016;150(5):1196-207.
5. Bianco AM, Zanin V, Girardelli M, Magnolato A, Martelossi S, Martellossi S, et al. A common genetic background could explain early-onset Crohn's disease. Med Hypotheses. 2012;78(4):520-2.
6. Avitzur Y, Guo C, Mastropaolo LA, Bahrami E, Chen H, Zhao Z, et al. Mutations in tetratricopeptide repeat domain 7A result in a severe form of very early onset inflammatory bowel disease. Gastroenterology. 2014;146(4):1028-39.
7. Kammermeier J, Drury S, James CT, Dziubak R, Ocaka L, Elawad M, et al. Targeted gene panel sequencing in children with very early onset inflammatory bowel disease--evaluation and prospective analysis. J Med Genet. 2014;51(11):748-55.
8. Salzer E, Kansu A, Sic H, Májek P, Ikincioğullari A, Dogu FE, et al. Early-onset inflammatory bowel disease and common variable immunodeficiency-like disease caused by IL-21 deficiency. J Allergy Clin Immunol. 2014;133(6):1651-9.e12.

9. Muise AM, Xu W, Guo CH, Walters TD, Wolters VM, Fattouh R, et al. NADPH oxidase complex and IBD candidate gene studies: identification of a rare variant in NCF2 that results in reduced binding to RAC2. Gut. 2012;61(7):1028-35.
10. Brigida I, Zoccolillo M, Cicalese MP, Pfajfer L, Barzaghi F, Scala S, et al. T-cell defects in patients with. Blood. 2018;132(22):2362-74.
11. Arnold DE, Heimall JR. A Review of Chronic Granulomatous Disease. Adv Ther. 2017;34(12):2543-57.
12. Wang XC. [Clinical features of X-linked agammaglobulinemia: analysis of 8 cases]. Zhonghua Er Ke Za Zhi. 2004;42(8):564-7.
13. Conley ME, Dobbs AK, Quintana AM, Bosompem A, Wang YD, Coustan-Smith E, et al. Agammaglobulinemia and absent B lineage cells in a patient lacking the p85α subunit of PI3K. J Exp Med. 2012;209(3):463-70.
14. Ferrari S, Lougaris V, Caraffi S, Zuntini R, Yang J, Soresina A, et al. Mutations of the Igbeta gene cause agammaglobulinemia in man. J Exp Med. 2007;204(9):2047-51.
15. Betterle C, Greggio NA, Volpato M. Clinical review 93: Autoimmune polyglandular syndrome type 1. J Clin Endocrinol Metab. 1998;83(4):1049-55.
16. Kelsen JR, Baldassano RN, Artis D, Sonnenberg GF. Maintaining intestinal health: the genetics and immunology of very early onset inflammatory bowel disease. Cell Mol Gastroenterol Hepatol. 2015;1(5):462-76.
17. Lübbehusen J, Thiel C, Rind N, Ungar D, Prinsen BH, de Koning TJ, et al. Fatal outcome due to deficiency of subunit 6 of the conserved oligomeric Golgi complex leading to a new type of congenital disorders of glycosylation. Hum Mol Genet. 2010;19(18):3623-33.
18. Huybrechts S, De Laet C, Bontems P, Rooze S, Souayah H, Sznajer Y, et al. Deficiency of Subunit 6 of the Conserved Oligomeric Golgi Complex (COG6-CDG): Second Patient, Different Phenotype. JIMD Rep. 2012;4:103-8.
19. van Zelm MC, Smet J, van der Burg M, Ferster A, Le PQ, Schandené L, et al. Antibody deficiency due to a missense mutation in CD19 demonstrates the importance of the conserved tryptophan 41 in immunoglobulin superfamily domain formation. Hum Mol Genet. 2011;20(9):1854-63.
20. Kanegane H, Agematsu K, Futatani T, Sira MM, Suga K, Sekiguchi T, et al. Novel mutations in a Japanese patient with CD19 deficiency. Genes Immun. 2007;8(8):663-70.
21. Kuijpers TW, Bende RJ, Baars PA, Grummels A, Derks IA, Dolman KM, et al. CD20 deficiency in humans results in impaired T cell-independent antibody responses. J Clin Invest. 2010;120(1):214-22.
22. van Zelm MC, Smet J, Adams B, Mascart F, Schandené L, Janssen F, et al. CD81 gene defect in humans disrupts CD19 complex formation and leads to antibody deficiency. J Clin Invest. 2010;120(4):1265-74.
23. Trotta L, Hautala T, Hämäläinen S, Syrjänen J, Viskari H, Almusa H, et al. Enrichment of rare variants in population isolates: single AICDA mutation responsible for hyper-IgM syndrome type 2 in Finland. Eur J Hum Genet. 2016;24(10):1473-8.
24. Sogkas G, Dubrowinskaja N, Bergmann AK, Lentes J, Ripperger T, Fedchenko M, et al. Progressive Immunodeficiency with Gradual Depletion of B and $CD4^+$ T Cells in Immunodeficiency, Centromeric Instability and Facial Anomalies Syndrome 2 (ICF2). Diseases. 2019;7(2).
25. Chiriaco M, Di Matteo G, Conti F, Petricone D, De Luca M, Di Cesare S, et al. First Case of Patient With Two Homozygous Mutations in. Front Immunol. 2019;10:130.
26. Lucas CL, Kuehn HS, Zhao F, Niemela JE, Deenick EK, Palendira U, et al. Dominant-activating germline mutations in the gene encoding the PI(3)K catalytic subunit p110δ result in T cell senescence and human immunodeficiency. Nat Immunol. 2014;15(1):88-97.

27. Lohr NJ, Molleston JP, Strauss KA, Torres-Martinez W, Sherman EA, Squires RH, et al. Human ITCH E3 ubiquitin ligase deficiency causes syndromic multisystem autoimmune disease. Am J Hum Genet. 2010;86(3):447-53.
28. Christiano AM, McGrath JA, Tan KC, Uitto J. Glycine substitutions in the triple-helical region of type VII collagen result in a spectrum of dystrophic epidermolysis bullosa phenotypes and patterns of inheritance. Am J Hum Genet. 1996;58(4):671-81.
29. Kelsen JR, Dawany N, Moran CJ, Petersen BS, Sarmady M, Sasson A, et al. Exome sequencing analysis reveals variants in primary immunodeficiency genes in patients with very early onset inflammatory bowel disease. Gastroenterology. 2015;149(6):1415-24.
30. Janecke AR, Heinz-Erian P, Yin J, Petersen BS, Franke A, Lechner S, et al. Reduced sodium/proton exchanger NHE3 activity causes congenital sodium diarrhea. Hum Mol Genet. 2015;24(23):6614-23.
31. Kobayashi K, Fujita K, Okino F, Kajii T. An abnormality of neutrophil adhesion: autosomal recessive inheritance associated with missing neutrophil glycoproteins. Pediatrics. 1984;73(5):606-10.
32. Kelsen JR, Dawany N, Martinez A, Grochowski CM, Maurer K, Rappaport E, et al. A de novo whole gene deletion of XIAP detected by exome sequencing analysis in very early onset inflammatory bowel disease: a case report. BMC Gastroenterol. 2015;15:160.
33. Weinstein EJ, Bourner M, Head R, Zakeri H, Bauer C, Mazzarella R. URP1: a member of a novel family of PH and FERM domain-containing membrane-associated proteins is significantly over-expressed in lung and colon carcinomas. Biochim Biophys Acta. 2003;1637(3):207-16.
34. Zhou Q, Wang H, Schwartz DM, Stoffels M, Park YH, Zhang Y, et al. Loss-of-function mutations in TNFAIP3 leading to A20 haploinsufficiency cause an early-onset autoinflammatory disease. Nat Genet. 2016;48(1):67-73.
35. Sunseri WM, Kugathasan S, Keljo DJ, Greer JB, Ranganathan S, Cross RK, et al. IBD LIVE Case Series--Case 3: Very Early-Onset Inflammatory Bowel Disease: When Genetic Testing Proves Beneficial. Inflamm Bowel Dis. 2015;21(12):2958-68.
36. Schubert D, Bode C, Kenefeck R, Hou TZ, Wing JB, Kennedy A, et al. Autosomal dominant immune dysregulation syndrome in humans with CTLA4 mutations. Nat Med. 2014;20(12):1410-6.
